# Whole-genome analysis of 5-hydroxymethylcytosine and 5-methylcytosine at base resolution in the human brain

**DOI:** 10.1186/gb-2014-15-3-r49

**Published:** 2014-03-04

**Authors:** Lu Wen, Xianlong Li, Liying Yan, Yuexi Tan, Rong Li, Yangyu Zhao, Yan Wang, Jingcheng Xie, Yan Zhang, Chunxiao Song, Miao Yu, Xiaomeng Liu, Ping Zhu, Xiaoyu Li, Yu Hou, Hongshan Guo, Xinglong Wu, Chuan He, Ruiqiang Li, Fuchou Tang, Jie Qiao

**Affiliations:** 1 Biodynamic Optical Imaging Center & Center for Reproductive Medicine, College of Life Sciences, Third Hospital, Peking University, Beijing 100871, P. R. China; 2 Key Laboratory of Assisted Reproduction, Ministry of Education, Beijing 100191, P. R. China; 3 Department of Neurosurgery, Peking University Third Hospital, Beijing 100191, China; 4 Department of Chemistry & Institute for Biophysical Dynamics, The University of Chicago, Chicago, IL, USA; 5 Ministry of Education Key Laboratory of Cell Proliferation and Differentiation, Beijing 100871, P. R. China; 6 Peking-Tsinghua Center for Life Sciences, College of Life Sciences, Peking University, Beijing 100871, P. R. China

## Abstract

**Background:**

5-methylcytosine (mC) can be oxidized by the tet methylcytosine dioxygenase (Tet) family of enzymes to 5-hydroxymethylcytosine (hmC), which is an intermediate of mC demethylation and may also be a stable epigenetic modification that influences chromatin structure. hmC is particularly abundant in mammalian brains but its function is currently unknown. A high-resolution hydroxymethylome map is required to fully understand the function of hmC in the human brain.

**Results:**

We present genome-wide and single-base resolution maps of hmC and mC in the human brain by combined application of Tet-assisted bisulfite sequencing and bisulfite sequencing. We demonstrate that hmCs increase markedly from the fetal to the adult stage, and in the adult brain, 13% of all CpGs are highly hydroxymethylated with strong enrichment at genic regions and distal regulatory elements. Notably, hmC peaks are identified at the 5′splicing sites at the exon-intron boundary, suggesting a mechanistic link between hmC and splicing. We report a surprising transcription-correlated hmC bias toward the sense strand and an mC bias toward the antisense strand of gene bodies. Furthermore, hmC is negatively correlated with H3K27me3-marked and H3K9me3-marked repressive genomic regions, and is more enriched at poised enhancers than active enhancers.

**Conclusions:**

We provide single-base resolution hmC and mC maps in the human brain and our data imply novel roles of hmC in regulating splicing and gene expression. Hydroxymethylation is the main modification status for a large portion of CpGs situated at poised enhancers and actively transcribed regions, suggesting its roles in epigenetic tuning at these regions.

## Background

Methylation of cytosine (mC) plays a role in many crucial cellular processes. In 2009, it was shown that mC can be oxidized to 5-hydroxymethylcytosine (hmC) by tet methylcytosine dioxygenase (Tet) family of enzyme, and that embryonic stem cells (ESCs) and mouse brain tissues contain high levels of hmC [1,2]. These and subsequent findings evidently suggested that hmC is an intermediate in the long pursued pathway of active DNA demethylation [3,4].

Soon after the discovery, a series of genome-wide mapping studies of hmC were performed using affinity or enzyme-based approaches by us and others [5-16]. These studies, at low resolution and semi-quantitative, have provided significant insights into the distribution and functions of hmC at distal regulatory elements, gene bodies, and polycomb repression complex-bound promoters. More recently, two bisulfite-sequencing (BS-Seq) derived methods, Tet-assisted bisulfite sequencing (TAB-Seq) and oxidative bisulfite sequencing (oxBS-Seq), were established to quantitatively sequence hmC at base resolution [17,18]. The first genome-wide application of TAB-Seq to mammalian ESCs revealed novel characters of hmC such as its deposition around, but not within, transcription factor binding sites [17].

The content of hmC in the mammalian brain is typically five to ten times higher than in any other tissues, suggesting a potential role for hmC in the brain [2,8,19]. hmC could be an intermediate of mC demethylation, suggesting a potential high turnover rate of DNA methylation in the brain [20]. In addition, given its high abundance and stability, hmC could act as an epigenetic modification that influences genome structure and function by recruiting chromatin modifiers [21,22]. Recent studies have shown that Tet1 mutant mice exhibit memory defects, suggesting that DNA hydroxymethylation plays an important role in normal brain function [23,24].

However, the exact function of hmC in the mammalian brain remains to be understood. Recently, Lister *et al.* reported comprehensive genome-wide DNA methylation maps in the human and mouse brain using BS-Seq, which also include hmC maps in the mouse brain using TAB-Seq [25]. Here, we applied TAB-Seq combined with BS-Seq to map the DNA hydroxymethylome and methylome at single-base resolution in the human brain. Our data uncovered new features of hmC including hmC peaks at 5′ splicing sites and a transcription-corrected hmC bias toward the sense strand of gene bodies, implying novel roles of hmC in regulating splicing and gene expression in the brain.

## Results and discussion

### Base-resolution hydroxymethylome and methylome mapping in the human brain and identification of highly hydroxymethylated cytosines

**Figure 1:**
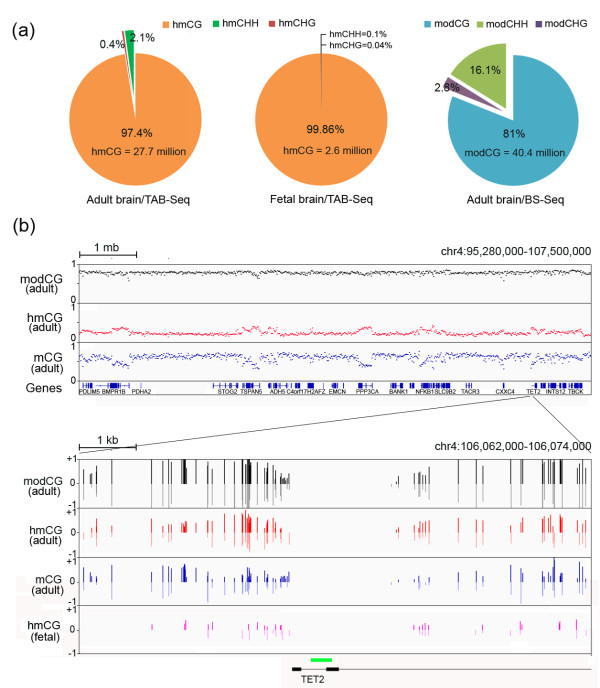
**Base-resolution hydroxymethylome and methylome in the human brain. (a)** The percentages of hmCs or modCs in the adult or the fetal brain in the contexts of CG, CHH, and CHG. **(b)** Examples of the hmC, mC, and total modification (hmC + mC) profiles are shown for a genomic region of 12 mb on chromosome 4 as a scatterplot (Upper panel) and for a 12 kb region surrounding the TSS of the *TET2* gene as a bar chart. The green box indicates the CpG island located in the *TET2* promoter.

We performed TAB-Seq and BS-Seq on a DNA sample isolated from the prefrontal cortex of an adult individual and sequenced it to an average depth of 22× per strand by TAB-Seq and 9.3× by BS-Seq. For TAB-Seq, we observed a low non-conversion rate of unmodified cytosine (0.36%) and mC (1.18%), and a high protection rate of hmC (97.6%). We also applied TAB-Seq to a DNA sample isolated from the prefrontal cortex of a fetal brain and sequenced it to an average depth of 11× per strand, with the non-conversion rates of unmodified cytosine and mC being 0.25% and 1.51%, respectively. The sequencing details are summarized in Additional file 1. TAB-Seq detected approximately 28.4 million hmCs in the adult prefrontal cortex, 10-fold more than that in the fetal prefrontal cortex (approximately 2.6 million), and BS-Seq detected approximately 49.9 million modified cytosines (modCs) (false discovery rate ≤1%; Figure 1A, and see Materials and methods). The much higher number of hmCs detected in the adult prefrontal cortex is consistent with previous reports [8,9]. To quantitatively verify this, we performed liquid chromatography-tandem mass spectrometry (LC-MS/MS) to genomic DNAs isolated from several regions of these two brain samples, as well as another pair of fetal and adult brain samples, and the results confirmed that the abundance of hmC in the adult human brain is nearly six times higher than that in the fetal brain (%hmC/dC average 0.866 *vs* 0.154) (Additional files 2 and 3). Whereas a notable portion of modC exists in non-CpG contexts in the adult prefrontal cortex (16.1% in CHH and 2.8% in CHG, where H = A, C or T), hmC exists predominantly in CpG context (97.4% in the adult cortex and 99.86% in the fetal cortex), which is in line with the finding in mouse [25] (Figure 1a). We also applied BS-Seq and TAB-Seq to a DNA sample extracted from the hippocampus of the fetal brain, and found that both modC and hmC exists predominantly in CpG context, confirming the previous finding that the non-CpG modification in the brain is adult-specific [25] (Additional file 4). Since the majority of hmC exists in a CpG context, we next focused our analysis on CpG modifications.

Examples of the hmC and mC maps were shown in Figure 1b. A genomic region of 12 mb (mega base pairs) on chromosome 4 displays increased hmC levels in gene-rich regions, coincident with a decrease in mC levels; these changes could not be distinguished by traditional bisulfite sequencing alone because the modC levels measured by BS-Seq change only slightly. Closer inspection of a 12 kb (kilo base pairs) region around the *TET2* transcription start site (TSS) further supports the need for TAB-Seq. For many individual CpG sites, the hmC level is higher than the mC level. In particular, the highly modified CpGs immediately upstream the TSS are highly hydroxymethylated and could be mistaken as highly methylated sites when applying traditional BS-Seq alone. These results highlight the need to apply TAB-Seq to distinguish hmC and mC unequivocally for obtaining accurate hydroxymethylation and methylation maps in the brain. The hmC map of the fetal brain is also provided and suggested that patterns of hmCs in the adult brain have been partially established in the fetal stage.

**Figure 2:**
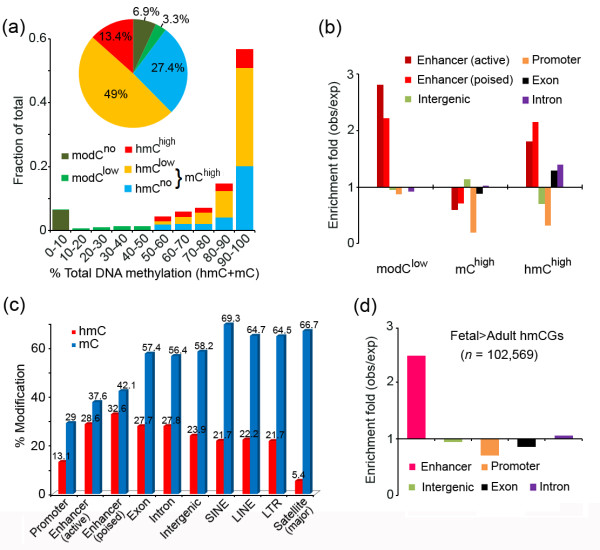
**Features of hydroxymethylome in the human brain. (a)** Classification of all CpGs in the adult brain according to their hydroxymethylation and methylation frequencies. **(b)** Fold enrichment of the CpG categories on different genomic elements. hmC^high^ is enriched in enhancers, exons and introns. **(c)** The absolute hmC and mC levels at different genomic elements in the adult brain. **(d)** Fold enrichment of the Fetal > Adult hmCGs, which exhibited higher hmC levels in the fetal brain than in the adult brain, on different genomic elements.

We calculated the modification frequency of hmC and mC for individual CpG sites. In general, hmCs occur at relatively low frequency (median 29.2% and 30.8% in the adult and fetal brains, respectively), whereas most mCs occur at high frequency (median 59.7% in the adult brain) (Additional file 5a and b). Then we classified CpG sites according to their hmC and mC frequencies. We first divided all CpGs into three categories depending on the total modification (hmC + mC, or modC). In agreement with previous studies [26], we determined that 89.8% of the CpGs are highly modified (modC^high^, modC ≥ 50%), 6.9% are unmodified (modC^no^, modC < 10%), and 3.3% (*n* = 1,423,006) are modified at low levels (modC^low^, 10% ≤ modC < 50%) (Figure 2a). Next, we divided the modC^high^ into three subgroups: (1) hmC^high^: hmC > mC; (2) hmC^low^: hmC < mC; and (3) hmC^no^: no hmC. We determined that hmC^high^ (*n* = 5,692,354) accounts for a notable proportion (13.4%) of all captured CpGs. The hmC^low^ (*n* = 20,857,810) and hmC^no^ (*n* = 11,695,002) categories comprised 49% and 27.4%, respectively. Since further analysis revealed that hmC^low^ and hmC^no^ have similar characters, they were grouped into mC^high^ referring to highly-methylated cytosines (Figure 2a).

### Genomic distribution of hmC in the human brain

Next, we determined the genomic distribution of modC^low^, hmC^high^, and mC^high^. In addition to the annotated genomic features, we also analyzed enhancers mapped by a recently published ChIP-Seq dataset in the human brain [27]. Two types of enhancers were distinguished: active enhancers that were simultaneously marked by distal H3K4me1 and H3K27ac, and poised enhancers that were solely marked by distal H3K4me1 [28]. modC^low^ is prominently enriched at enhancers (Figure 2b), which is in accordance with previous bisulfite studies [26]. We found that hmC^high^ is also highly enriched at enhancers. In contrast to more enrichment of modC^low^ at active enhancers, hmC^high^ is more enriched at poised enhancers (Figure 2b). Furthermore, hmC^high^ is abundant at introns and exons. Most hmC^high^ (56.9%) occur at genic regions (Additional file 5c), and this is distinct from hmC distribution in ESCs [17]. By contrast, mC^high^ are not enriched at enhancers or genic regions. We calculated the hmC and mC levels of these genomic elements (Figure 2c). We observed that poised enhancers have the highest hmC level (32.6% on average), followed by active enhancers (28.6%), introns (27.8%), exons (27.7%), and intergenic regions (23.6%). The mC levels follow the reverse order, with the intergenic regions (58.2%) being highest, followed by exons (57.4%), introns (56.4%), and enhancers (42.1% and 37.6% for poised and active enhancers, respectively). The promoter regions have both the lowest hmC and mC levels. Repetitive sequences, including LINE, SINE, LTR, and major satellite repeats, generally have lower hmC and higher mC levels than the non-repetitive regions, and the major satellite repeats have the lowest hmC level (5.4%).

We determined that the hmC levels of the fetal brain are much lower than those of the adult brain in all genomic regions (Additional file 5d). Despite this, we identified 102,569 CpGs that exhibited higher hmC levels in the fetal brain than in the adult brain (both coverage ≥10, methylation difference ≥0.3). Analysis for the genomic distribution of these Fetal > Adult hmCGs showed that they are highly enriched at enhancers, but not at other genomic features (Figure 2d). The result suggested that hmC changes at enhancers are bidirectional with a subset of enhancers gaining and some losing hydroxymethylation from the fetal to the adult stage, which is consistent with the previous report that hmC marks regulatory elements in the fetal brain that is poised for subsequent activation in the adult brain [25].

### Prominent hmC peaks mark the exon-intron boundary

**Figure 3:**
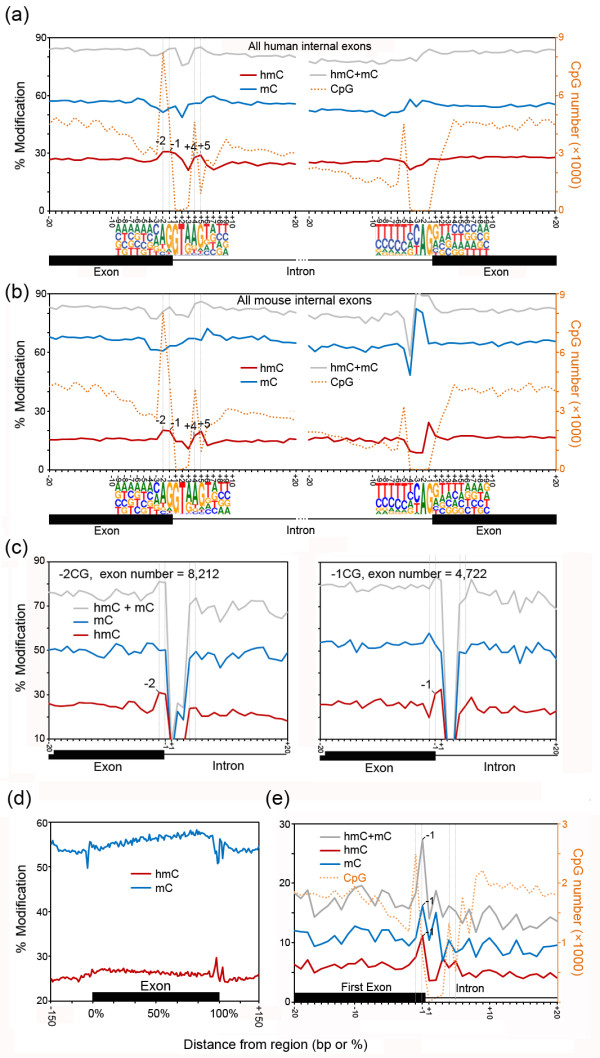
**Prominent hmC changes mark the exon-intron boundary. (a, b)** Profiles of hmC and mC for a 40-bp window around the exon-intron and intron-exon boundaries at single-nucleotide resolution in human (a) and mouse (b). Modification levels of hmC, mC, total DNA methylation (hmC + hmC), and the CpG number are shown for all internal exons in the sense strand. The sequences ± 9 bp around the 5′ and 3′ splicing sites are also indicated. The TAB-Seq and BS-Seq data to generate the mouse profile (b) were obtained from Lister *et al.*[25]. **(c)** Profiles of hmC and mC at the exon-intron boundary of exons which have a CpG at 5′ss position -2 (-2CG, *n* = 8,212) or -1 (-1CG, *n* = 4,277). Since a CpG at one position will lead to absence of CpG at the nearest neighboring position and thus no methylation value, we merged the data of the sense and the antisense strands for each type of exons. **(d)** Profiles of hmC and mC across exons. All internal exons were divided into 100 bins, and average hmC and mC levels were calculated for each bin, as well as ±150 bp surrounding the exon. **(e)** The hmC and mC profiles at the exon-intron boundary of the first exon.

Despite previous suggestions that hmC may be associated with the exon-intron boundary in the brain [14], the low-resolution method employed previously prevented accurate analysis at the base resolution. Conversely, whole-genome BS-Seq studies have shown that DNA modification is abundant on exons [29-31]. These studies, however, were not able to distinguish between mC and hmC. One intriguing finding of our analyses of the base-resolution hmC and mC maps is two striking hmC peaks at the 5′ splicing sites (5′ss), which are positioned flanking the highly conserved ‘GT’ sequence, with a higher peak siting at positions -1 and -2 on the exon side and a lower one at positions +4 and +5 on the intron side (all internal exons, *n* = 176,455) (Figure 3a and Additional file 6). In contrast, mC is not enriched. Notably, analysis of the recently published TAB-Seq (GSM1173795) and BS-Seq (GSM1173783) data generated from the adult mouse brain tissue (frontal cortex from 6-week-old male mouse brain) [25] revealed two nearly identical hmC peaks at the 5′ss, indicating that this pattern is conserved in mammals (Figure 3b). The CpGs at both peaks are not part of the consensus 5′ss sequence (CAG/GTAAGT). However, the peak positions -2 and +4 have higher tendencies to be CpG sites than the overall exons and introns (Figure 3a and b). The profile of CpG distribution has been recently reported [32]. We further determined that, of the 176,455 examined human exons, 12,934 (7.3%, 4722 at position -1 and 8,212 at position -2) have CpG sites at positions related to the exon-side peak and 5,514 (3.1%, 4605 at position +4 and 909 at position +5) have CpG sites related to the intron-side peak (Figure 3a). Overall, these exons (*n* = 18,036, 10.2% of all 176,455 human internal exons) represent a large proportion of human genes (*n* = 9,103, 48.9% of all 18,606 human genes, see Additional file 7 for 5′ss sequences of these exons and the corresponding genes). To exclude the possibility that the hmC peaks are artifacts due to higher hmC levels of the exons which have a CpG site at the 5′ss than those do not have, we separately analyzed the exons which have a CpG site at the 5′ss position -2, -1, +4 or +5, and the results are similar to that for all internal exons (Figure 3c and Additional file 8). These results together indicated that prominent hmC peaks mark the 5′ss at the exon-intron boundary in both human and mouse, and are associated with a large cohort of internal exons.

A decrease in hmC at positions +6 and +7 directly following the intron-side hmC peak was also observed for all internal exons (Figure 3a and Additional file 5: Figure S4), which seems to be similar to the intronic hmC decrease reported previously [14]. In addition to these hmC changes at the 5′ss, we also observed a marked increase in mC and a less pronounced decrease in hmC from 5′ to 3′ across the exons (Figure 3c). We also examined the first exons (*n* = 12,980), and found that they have higher CpG occurrence and much lower hmC and mC levels than the internal exons (Figure 3d), which should be due to their proximity to promoter CpG islands. Interestingly, in contrast to the internal exons, mC is strongly enriched, and hmC change follows the mC change. The distinction between the internal exons and the first exons is suggestive of a functional connection between the hmC peaks and alternative exon inclusion, which should only occur in the internal exons.

**Figure 4:**
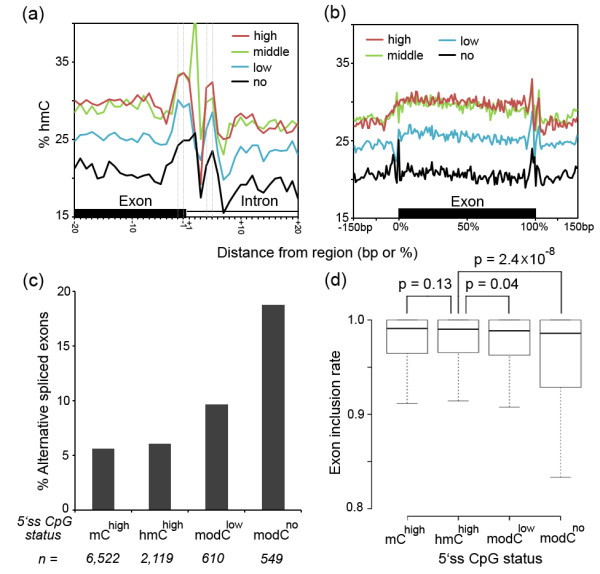
**Correlation of the hmC and mC changes at the exon-intron boundary with gene expression and splicing. (a, b)** The hmC changes at the exon-intron boundary (a) and across the exon (b) are similar for exons expressed at high (red), middle (green), or low (blue) levels, as well as exons with no expression (black). **(c, d)** The percentages of alternatively spliced exons (c) and the inclusion rates (d) of the exons with the status of the 5′ss CpG site being mC^high^, hmC^high^, modC^low^, or modC^no^ showing differential splicing of these exon types. Two-tailed MWW test. *n*, number of exons.

To determine whether the observed hmC and mC changes are associated with gene expression, we generated RNA-Seq data using RNA extracted from the same adult brain sample for TAB-Seq and BS-Seq and divided the expressed genes (RPKM >0.1) into three groups of identical size of high, middle and low expression levels (Additional file 9). The genes within these three groups, as well as genes not expressed, displayed similar hmC changes around their exon-intron boundary. This was unlike overall hmC levels within both exons and introns, which positively correlates to gene expression levels (Figure 4a). In addition, the hmC decrease and the mC increase across the exon are also similar among genes transcribed at different levels (Figure 4b). These results suggested that the hmC and mC changes occur irrespective of the transcription levels of the corresponding genes.

Next, we address the potential functional relationship between the DNA modification at the exon-intron boundary and the splicing. We classified the exons having a CpG site at the 5′ss position -2, -1, +4 or +5 into four types according to the DNA modification states of the CpG site (that is, mC^high^, hmC^high^, modC^low^, or modC^no^) and compared their inclusion rates (see Materials and methods). We also examined the number of the alternatively spliced (AS) exon, which was defined as the inclusion rate being less than 0.8. The results demonstrated that the hmC^high^ and mC^high^ exons comprised a similar fraction of AS exons (6.04% (128 out of 2,119 exons) *vs.* 5.63% (367 out of 6,522 exons), Figure 4c), with the inclusion rates being no difference (median 0.9901 *vs.* 0.9908, *P* = 0.13, two-tailed Mann-Whitney-Wilcoxon (MWW) test, Figure 4d). Also no difference was found comparing the hmC^high^ and mC^high^ exons with the internal exons put together (median inclusion rate 0.9912, *P* >0.1, AS exons percent: 6.76% (6,929 out of 102,474 exons)). These results suggested that hmC and mC at the 5′ss CpG site are similarly related to splicing. Interestingly, the modC^low^ and modC^no^ groups involved markedly more AS exons (9.67% (59 out of 610 exons) and 18.76% (103 out of 549 exons), respectively, Figure 4c). In addition, the inclusion rates of the modC^low^ and modC^no^ exons were slightly but significantly reduced comparing with the hmC^high^ and mC^high^ groups (modC^low^*vs.* hmC^high^: median 0.9885 *vs.* 0.9901, *P* = 0.04; modC^no^*vs.* the hmC^high^: median 0.9859 *vs.* 0.9901, *P* = 2.45 × 10^-8^, two-tailed MWW test, Figure 4d). These data suggested that complete demethylation of mC and hmC to C might lead to exon skipping, which is in line with previous studies that DNA methylation facilitates exon recognition [32,33].

### Strand-biased hmC and mC on the gene body

**Figure 5:**
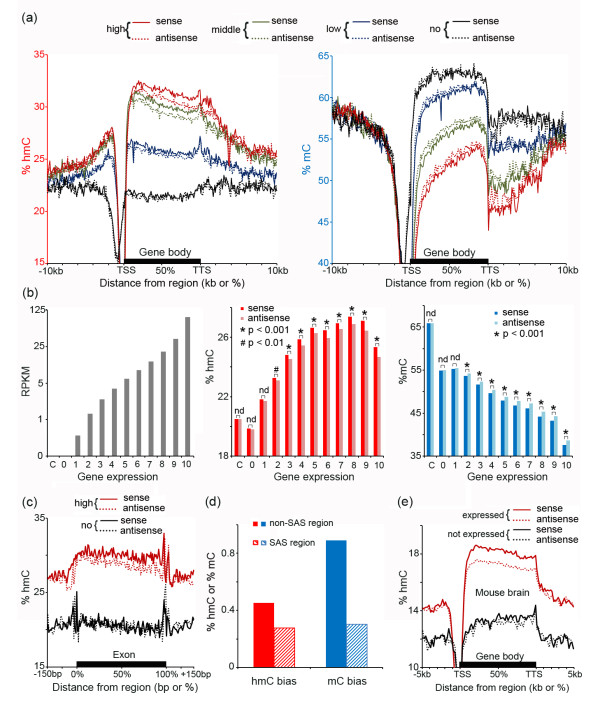
**Strand-biased hmC and mC profiles on the gene body. (a)** Profiles of hmC (left panel) and mC (right panel) on the sense (lined) and antisense (dotted) strands of the genes expressed at high (red), middle (green) and low (blue) expression levels, as well as the genes with no expression (black). TSS and TTS indicate the transcription starting site and the transcription terminal site, respectively. **(b)** Expressed genes were divided into 10 groups according to the expression levels (left panel), and the average levels of hmC (middle panel) and mC (right panel) for each strand of the gene body were measured. The values for the genes that are not expressed (expression level 0) and randomly selected intergenic regions as the control (C) are also shown. One-tailed paired Student’s *t* test. nd, no statistical difference (*P* >0.05). **(c)** Profiles of hmC on the sense (lined) and antisense (dotted) strands of exons with high (red) or no (black) expression. **(d)** The hmC and mC strand-biases are reduced at the sense-antisense gene (SAS) paired regions in comparison with the non-SAS regions. **(e)** The hmC profile on the sense (lined) and antisense (dotted) strands of the genes that are expressed (red) or not expressed (black) in the mouse brain exhibits the transcription-correlated hmC bias toward the sense strand similar to the human pattern. The TAB-Seq, BS-Seq, and RNA-Seq data for analysis were obtained from Lister *et al.*[25].

Previous studies have reported that hmC is enriched on gene bodies and positively correlated with gene expression in the adult brain [8,9,13,21,25]. We plotted the average hmC and mC levels across the genes at high, middle, and low expression levels and the data quantitatively confirmed that hmC is markedly enriched on the gene body and positively correlated with gene expression levels in the human adult brain (Figure 5a, left panel). In addition, we also showed that mC levels are clearly negatively correlated with gene expression (Figure 5a, right panel). Surprisingly, we observed that the hmC and mC abundances are slightly but significantly different between the sense and antisense strands, with enrichment of hmC on the sense strand and enrichment of mC on the antisense strand. The differences are positively correlated with gene expression levels as highly expressed genes show the strongest biases, whereas genes that are not expressed exhibit nearly no difference (Figure 5a).

To evaluate this pattern quantitatively and at a higher level of resolution, all of the expressed genes were divided into 10 equal-sized groups (approximately 1,400 genes for each group) based on their expression levels and the gene-body mC and hmC levels were calculated for each strand of the genes. The results revealed that, from the lowest to highest expressed genes, an average seven-fold increase in the hmC bias (%hmC of the sense strand minus that of the antisense strand, from 0.09% to 0.69%) and an average five-fold increase in the mC bias (%mC of the antisense strand minus that of the sense strand, from 0.22% to 1.04%) were observed (Figure 5b). Also, the strand differences of both hmC and mC are significant (for example, *P* = 1.63 × 10^-23^ for hmC and *P* = 4.4 × 10^-13^ for mC in genes of express group 9, two-tailed and paired Student’s *t*-Test, see Additional file 10 for all calculated *P* values, as well as the hmC, mC, and modC levels on each strand of all individual genes). Both hmC and mC also displayed a slight difference in genes that are classified as not expressed (*n* = 8,789), but these differences were not statistically significant (*P* = 0.39 for hmC and *P* = 0.44 for mC). No strand difference was found for randomly-selected intergenic regions (Figure 5b). Also, for most expression levels, the strand differences of the total modification were not significant as measured by BS-Seq (Additional file 11), explaining why it has not been observed in previous studies using BS-Seq alone.

Then we plotted the average hmC and mC levels across each strand of the exon and the results showed that the exon exhibited the strand difference at a similar level as that of the gene body, suggesting that the DNA methylation strand difference is a general feature of the transcribed exons and introns (Figure 5c and Additional file 12). To further verify the strand-bias, we analyzed the sense-antisense (SAS) gene paired regions. Since within these regions, each strand serves as both sense and antisense strands, it is expected that the strand difference should be lessened. The results indeed showed that both the hmC and mC biases decreased in the SAS regions, with a 39% reduction in the hmC bias (0.45% in non-SAS region *vs.* 0.28% in SAS region) and a 66% reduction in the mC bias (0.89% in non-SAS region *vs.* 0.3% in SAS region, Figure 5d). To generalize our observation, we analyzed the recently published TAB-Seq and BS-Seq data in the adult mouse brain [25]. The results notably showed that the mouse brain cells share a similar transcription-associated hmC bias toward the sense strand (Figure 5e) and an mC bias toward the antisense strand (Additional file 13), indicating that these hmC and mC features are evolutionary conserved between mouse and human.

**Figure 6:**
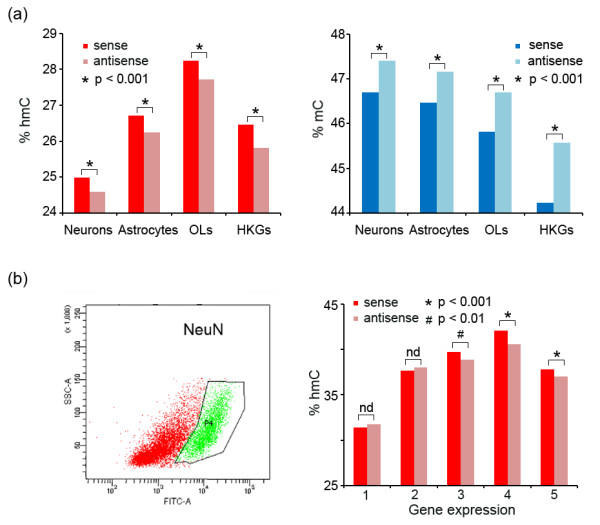
**Strand-biased hmC and mC profiles in both neurons and glia. (a)** The average levels of hmC (left panel) and mC (right panel) for each strand of genes enriched in different brain cell types including neurons, astrocytes and oligodendrocytes (OLs), as well as the house-keeping genes (HKGs). **(b)** Tet-assisted reduced representation bisulfite sequencing performed in neuron nuclei isolated by FACS using the NeuN antibody (left panel) revealed that hmC is significantly enriched on the sense strands of the neuronal genes expressed at high (3, 4, 5), but not low (1, 2) expression levels (right panel). One-tailed paired Student’s *t* test. nd, no statistical difference (*P* >0.05).

To determine whether the hmC and mC strand biases are cell type-specific, we examined genes enriched in different brain cell types including neurons (*n* = 1,417), astrocytes (*n* = 1,807) and oligodendrocytes (*n* = 1,453) [34]. We found that all three gene sets and the house-keeping genes (*n* = 2,402) [35] exhibited enrichment of hmC on the sense strand and enrichment of mC on the antisense strand (*P* <0.001, Figure 6a). Furthermore, to independently validate our findings, we isolated neuronal nuclei by FACS (Figure 6b and Additional file 14) and employed a Tet-assisted reduced representation bisulfite sequencing (TA-RRBS) approach by performing TAB-Seq on MspI-enriched DNA fragments of genomic DNAs extracted from these neuronal nuclei, and then quantified the hmC levels on both stands of the neuron-enriched genes. The results confirmed that hmC is significantly enriched on the sense strands of the neuronal genes at high expression levels (for example, *P* = 2.4 × 10^-7^ for expression level 4, Figure 6b). The strand difference is not significant for genes expressed at low levels (*P* >0.05 for expression levels 1 and 2, Figure 6b). Together, our data indicated a transcription-correlated hmC bias toward the sense strand and an mC bias toward the antisense strand of the gene body in both neurons and glia in the human and mouse adult brain.

### hmC is enriched at poised enhancers and is negatively correlated with repressive histone modifications

**Figure 7:**
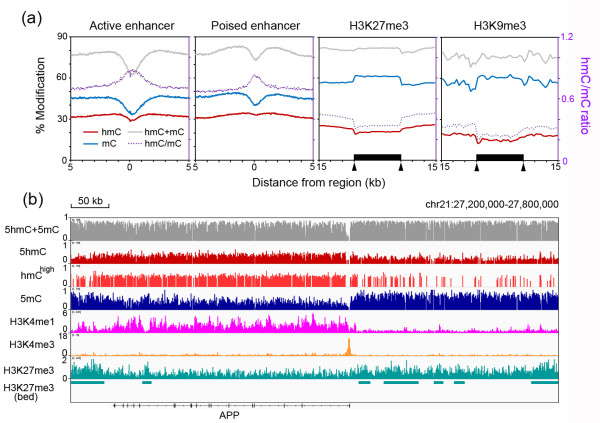
**hmC/mC profiles on enhancers and repressive genomic regions. (a)** The hmC, mC, and total DNA methylation levels, and the hmC/mC ratios were displayed surrounding the midpoints of active or poised enhancers, or across H3K27me3- and H3K9me3-enriched regions. Arrows indicate the starting and ending points of the H3K27me3- and H3K9me3-marked regions. **(b)** An example of a 600-kb genomic region surrounding the *APP* gene showing enrichment of hmC and H3K4me1 within the genic region and enrichment of mC and H3K27me3 in the neighboring intergenic regions. ChIP-Seq data for H3K4me1, H3K4me3, and H3K27me3 were obtained from Zhu *et al.*[26].

Next, we associated the hmC/mC profiles with the ChIP-seq data of various chromatin modification marks [27]. At the active enhancer, both hmC and mC levels are depleted towards the core region, and hmC is relatively accumulated at the flanking region. At the poised enhancer, both modifications revealed less pronounced reduction at the core region, particularly for hmC (Figure 7a). The difference in hmC profiles between the active and poised enhancers is more strikingly displayed by the high proportion of the hmC^high^ subgroup at the core region of the poised enhancer (Additional file 15). These results are consistent with previous reports in ESCs [15,17] and in the mouse brain [25], suggesting that hmC also participates in maintaining a ‘poised’ chromatin structure of enhancers in the human adult brain.

Lastly, we address the relationship between DNA modification and two repressive histone modifications, H3K27me3 and H3K9me3. The results demonstrated that hmC is clearly depleted at these repressive regions, with the average hmC levels of both the H3K27me3 and H3K9me3 regions being lower than the overall intergenic regions (20.5% *vs.* 23.9% and 14.6% *vs.* 23.9%, respectively, Figure 7a). By contrast, the mC levels in both of these repressive regions are higher than at the overall intergenic regions (approximately 64.4% *vs.* 58.2%, Figure 7a). An example of depletion of hmC on the genomic regions marked by H3K27me3 and H3K9me3 was provided in Additional file 16. It was shown recently that brain tissues exist in a unique chromatin state, with expansion of H3K27me3 over a particularly large proportion of the intergenic regions, accompanied by a dramatic restriction of H3K4me1 sites within the transcriptional units [27]. The association of our hmC/mC profiles with these two histone modification maps, as well as H3K4me3 peaks at the promoter regions, can be viewed in genomic regions surrounding the amyloid precursor protein (*APP*) and Neurexin 1 (*NRXN1*) gene loci as examples (Figure 7b and Additional file 17), which are representative of transcribed genes in the adult brain.

## Conclusion

In this study, we mapped DNA hydroxymethylation and methylation across the whole genome at single-base resolution in the human brain by combined application of TAB-Seq and BS-Seq, which revealed several novel insights regarding the nature of DNA methylation in brain tissues. The first intriguing finding of this study is the two striking hmC peaks at the 5′ss, that is, the sharp increase in hmC levels at positions -1 and -2 at the exon side and at positions +4 and +5 at the intron side surrounding the highly conserved ‘GT’ splicing sequence. A previous study has revealed enrichment of DNA modification (hmC + mC) at the exon-intron boundary in human embryonic stem cells and fibroblast [30]. Since BS-Seq alone was applied in this study, mC and hmC were not distinguished. In the present study, by the combined use of TAB-Seq and BS-Seq, we unexpectedly found that it is the hmC that forms peaks at the 5′ss in the adult human brain whereas mC is not enriched. This finding suggested that hmC is the key modification marking the exon-intron boundary in the adult brain tissues. It deserves to be mentioned that this pattern is evolutionary conserved since the mouse brain cells share similar hmC and mC features at the exon-intron boundary. A recent study also revealed significant hmC but not mC changes at the exon-intron boundary in the human and mouse brain. However, using an enzymatic method which recognized hmCs in the context of C^hm^CGG, this study only detected the intronic hmC decrease, while the more significantly marked hmC peaks were not reported [14]. Further studies are needed to elucidate how hmC peaks at the 5′ splicing sites are mechanically linked to splicing, which is under regulation by chromatin structures [36]. The results may indicate a high turnover rate of DNA methylation at the 5′ss, or that the hmC specifically recruits reader proteins that directly or indirectly affect splicing. Interestingly, our analysis suggested that both hmC and mC may facilitate exon recognition. This may be related with the reports that MeCP2 (methyl-CpG-binding protein 2), an extremely abundant protein in brain, binds to hmC and mC with similar high affinities and helps exon recognition [21,33].

Second, our data revealed a surprising transcription-correlated hmC bias toward the sense strand and an mC bias toward the antisense strand of the gene body in the adult brain tissues of both human and mouse. In contrast to the well-established role of DNA methylation in CpG island promoter regions for transcription repression, the function of gene body methylation is still largely unknown [37-41]. Previous studies have revealed a positive correlation between gene expression and the gene-body mC level [39-42]. More recently, we and others have shown the gene-body hmC level is also positively correlated with gene expression in brain tissues and germ line cells [8,9,16,21,25]. While it has been hypothesized that the function of gene-body methylation is to repress spurious initiation of transcription within active genes including that from the repetitive elements [41,43], our finding implies to an intrinsic link between the transcription elongation and the DNA modification processes. Interestingly, an enrichment of non-CG methylation on the antisense strand has been observed in ESCs previously [29]. Regarding the establishment of this strand bias, one possibility is that the antisense strand acts as the template for mRNA transcription; the non-template strand might thus be more accessible to TET proteins in some way, resulting in the enrichment of hmCG and, hence, the accumulation of mCG and non-CG methylation on the template strand. The asymmetry distribution of DNA hydromethylation and methylation might also be interpreted in brain cells to play some regulatory roles in transcription, which should need further functional studies in the future.

Third, we demonstrated that, in the adult human brain, 13.4% of all CpGs (*n* = 5,692,354) are highly-hydroxymethylated (the hmC^high^ category). Since hydroxymethylation is the main modification status of these CpG sites, it points to a regulatory role of these CpG sites when hydroxymethylated. Indeed, we found that this CpG category is strongly enriched at enhancers and genic regions, which is distinct from the highly-methylated (mC^high^), as well as the lowly-hydroxymethylated (hmC^low^, data not shown) categories. The hmC^high^ is also more enriched at poised enhancers, which is distinct from enrichment of modC^low^ at active enhancers. Finally, we showed that the hmC level is greatly reduced in H3K27me3-marked regions, which is different from its enrichment on the H3K27me3-associated bivalent promoters in ESCs [5-7]. This difference should be associated with the dramatic expansion of H3K27me3 in differentiated cells and tissues including the brain [27]. It will be of interest to investigate how these highly-hydroxymethylated CpG sites might contribute to the regulation of the unique chromatin organization in the brain tissues.

In summary, we present the genome-wide and single-base-resolution maps of hmC and mC in the human brain. The results imply novel roles for hmC in gene splicing and gene expression regulation. The identified highly-hydroxymethylated CpGs, which comprise 13.4% of all CpGs in human genome and are strongly enriched at poised enhancers and actively transcribed regions, also serve as a starting point for future investigations on the mechanisms that establish hydroxymethylation patterns and their associated functions in the human brain.

## Materials and methods

### Biological samples and genomic DNA extraction

This project was approved by the Reproductive Ethics Committee of Peking University Third Hospital (2012SZ010). Brain tissues for research were obtained with the written informed consent. The methods applied in this study are complied with the Helsinki Declaration. For TAB-Seq and BS-Seq, the adult human brain samples were obtained from a postmortem 42-year-old woman, and the fetal brain samples were obtained from an aborted 22-week-old male fetus. For LC-MS/MS, another adult human brain sample was obtained from a postmortem 28-year-old man, and another fetal sample was obtained from an aborted 22-week-old female fetus. All individuals had no sign of brain diseases. Genomic DNAs were extracted using Blood & Cell Culture DNA Kits (Qiagen) following the manufacturer’s instructions.

### Preparation of genomic DNA for TAB-Seq

For TAB-Seq, genomic DNAs and spike-in control DNAs were sheared to an average size of 200 bp using a Covaris S2 instrument. Glycosylation and oxidation of genomic DNAs was performed following a previous protocol with small modifications [44]. Briefly, 5 μg of sheared genomic DNA or with spike-in controls was initially glycosylated using β-glucosyltransferase proteins that were expressed and purified as previously described. Then, the DNA was purified using the QIAquick Nucleotide Removal Kit (Qiagen). Next, the oxidation reaction was performed using 1.5 μg of glycosylated DNA and 30 μL recombinant mTet1 protein in a 150 μL reaction solution and incubated for 1 h at 37°C. After proteinase K treatment, the oxidized DNA was first purified with Micro Bio-Spin 30 Columns (Bio-Rad) and then with 1.8 × Ampure XP Beads (Beckman) following the manufacturer’s suggestions.

For Tet-assisted reduced representation bisulfite sequencing (TA-RRBS), genomic DNAs extracted from purified neuron nucleus were first digested with MspI for 3 h at 37°C and purified using the QIAquick Nucleotide Removal Kit as described [45]. Then, glycosylation and oxidation of the DNA was performed similar to the TAB-Seq.

### Library preparation for TAB-Seq, BS-Seq, and sequencing

The DNA sample for BS-Seq was the same batch of sheared genomic DNA with spike-in controls that was used for TAB-Seq but without glycosylation and oxidation preparation. DNA samples for TAB-Seq and BS-Seq (0.5 to 1 μg) were end-repaired, A-tailed, and ligated to methylated adaptors using Illumina TruSeq Sample Preparation Kits following the manufacturer’s instructions. The ligated fragments were then bisulfite converted using the MethylCode Kit (Invitrogen). Bisulfite-treated DNA was separated into three reactions for PCR amplification using PfuTurbo Cx Hotstart DNA polymerase (Agilent Technologies) for seven cycles, yielding three independent libraries for the same biological sample. Final libraries were purified with 1× AMPure XP beads and sequenced using an Illumina Hiseq 2000. For Tet-assisted reduced representation bisulfite sequencing, an additional size selection of 180 to 600 bp fragments on a 2% agarose gel was performed after the adapter ligation.

### Spike-in controls for TAB-Seq, TARRBS, and BS-Seq

Fully methylated Lambda DNA was added to the human brain genomic DNAs in a ratio of 1 to 200 as a spike-in DNA control to calculate the non-conversion rates of unmodified cytosine and mC. For the spike-in control to calculate the non-conversion rates of hmC, a DNA fragment of approximately 1.6 kb was amplified from a pUC19 vector using a primer pair (5′-GCAGATTGTACTGAGAGTGC-3′ and 5′-TGCTGATAAATCTGGAGCCG-3′) and a cocktail of dATP/dGTP/dTTP and dhmCTP (Zymo Research, Cat. No. D1045). The control was then added to genomic DNA at a ratio of 1 to 400.

### RNA preparation and sequencing

Total RNA was extracted from the tissue sample using QIAGEN RNeasy Mini Kits (Qiagen), and mRNA was isolated by Sera-Mag Oligo (dT) beads (Thermo Scientific). Libraries were prepared using the NEB Next mRNA Sample PreP Master Mix Set 1 according to the manufacturer’s protocol and sequenced using an Illumina Hiseq 2000.

### Quantitative analysis of hmC using LC-MS/MS

Genomic DNA (2 μg) was digested by nuclease P1, venom phosphodiesterase I (Type VI), and alkaline phosphatase (Sigma). After desalting and filtration, 10 μL of the solution was injected into HPLC-MS/MS for analysis. HPLC-MS/MS was carried out by reverse-phase ultra-performance liquid chromatography on an Agilent ZORBAX Eclipse XDB-C18 column (Rapid Resolution HT, 50 × 2.1 mm P.N. 927700-902), equipped with a ZORBAX Eclipse XDB-C8 guard column (Column: P.N. 821125-926, Cartridges P.N. 820555–901), eluted with buffer A (0.1% formic acid in H2O) and buffer B (0.1% formic acid in methanol) with a flow rate of 0.5 mL min-1 at 35°C with a 2% to 25% gradient in 4.5 min, with online mass spectrometry detection using Agilent 6410 triple-quadrupole (QQQ) LC mass spectrometer in multiple reaction monitoring (MRM) positive electrospray ionization (ESI) mode. The nucleosides were quantified using the nucleoside-to-base ion mass transitions of 258 to 142 with collision energy of 1 eV (hmC) and 228 to 112 with collision energy of 5 eV (C). Quantification and detection limits were determined by comparison with the standard curves obtained from nucleoside standards running at the same volume and time.

### Neuronal nuclei isolation

Nuclei extraction was processed as described [46]. Briefly, 500 mg of cryopreserved human left frontal cortex was homogenized on ice by douncing in 5 mL lysis buffer (0.32 M Sucrose, 5 mM CaCl2, 3 mM, 0.1 mM EDTA, 10 mM Tris-HCl (pH 8.0), 1 mM DTT, and 0.1% TritonX-100). Homogenates were transferred to ultracentrifuge tube, with Sucrose Solution (1.8 M Sucrose, 3 mM Mg(Ac)2, 1 mM DTT, and 10 mM Tris-HCl (pH 8.0)) carefully pipetted at the bottom of the tube to form a concentration gradient. Ultracentrifugation was performed at 100,000 g for 2.5 h at 4°C. After centrifuge, supernatant including debris was removed and the pellet was incubated in 0.5 mL cold PBS on ice for 20 min before thoroughly triturated by pipetting.

Immunostaining mix was prepared by combining 300 μL PBS, 1.2 μg NeuN antibody (Millipore, MAB377), 200 μL Blocking Mix ( 2.5% BSA and 10% Goat Serum in PBS), and 2 μg of Alexa Fluor 488 conjugated secondary antibody (Cell Signaling, #4488) together and rotated for 5 min at room temperature in the dark. An isotype antibody control was processed in parallel by adding the same amount of mouse IgG instead of NeuN antibody. Then 1 mL nuclei suspension was added, and the mixture was incubated by rotating in the cold room for 45 min. After incubation, samples were retrieved and stained with Hoechst 33342 for another 2 min. The immunostaining result was checked under the microscope.

Immumotagged samples were diluted 10 times in PBS and filtered through a 40 μm filter before loaded to the FACS machine. A preliminary run was performed to gate out the proper nuclei size and the fluorescence bright population before the sort which separate the NeuN + nuclei. Once the sort was done, a small amount of sorted sample was run again through the instrument to confirm the purity.

After FACS, PBS was added to raise the volume of the sort to 10 mL. Then the sorted sample was mixed with 2 mL Sucrose Solution, 50 μL CaCl2 (1 M), and 30 μL Mg(Ac)2 (1 M), and incubated on ice for 15 min before centrifuged at 1,786 g for 15 min at 4°C. The NeuN + nuclei pellet was resuspended in PBS and went on with the Tet-assisted reduced representative bisulfite sequencing.

### Data processing

FASTQ format reads generated by the Illumina HiSeq2000 platform were aligned to the human reference sequence (HG19) using the Bismark program. Briefly, first, whole or any subsets of adaptor sequences were trimmed on 3′ and 5′ of reads before alignment. Second, a read was removed if more than 10% bases were N, or more than 50% bases were of Phred quality lower than 5, or at least three unmethylated CHs were present, or PCR redundancy occurred, so that only high quality data were used for downstream analysis, as described previously [17,29]. Third, cytosines in a read were computationally replaced with thymines and then mapped to computationally converted HG19 references using the Bismark program. The Lambda genome and the pUC19 sequences for the hmC spike-in control were also included in the reference sequence as extra chromosomes for assessing the non-conversion rates of unmodified cytosine, mC, and hmC.

### Assessing the non-conversion rates of unmodified cytosine, mC, and hmC and the protection rate of hmC

The non-conversion rate of unmodified cytosine was calculated as the percentage of sequenced cytosines in non-CG contexts relative to all covered cytosines in non-CG contexts in the Lambda genome. The non-conversion rate of mC was calculated as the value in a CG context, and the non-conversion rate of hmC was measured as the value for all cytosines in the spike-in pUC19 sequences. The original data are shown in Additional file 1: Table S1. The normalized protection rate of hmC was assessed by dividing the non-conversion rate of hmC of TAB-Seq by that of BS-Seq.

### Assessing the false discovery rates of modC and hmC

The *P* value for each cytosine detected by TAB-Seq and BS-Seq was calculated *via* binomial distribution as previously described [17,29]. To measure a false discovery rate (FDR) for the modC and hmC sites passing a given *P* value cutoff, we applied the Benjamini-Hochberg method [47] and set the FDR <0.01. The FDR was calculated separately for each chromosome and cytosine in each of the CG, CHG, and CHH contexts. We also applied the method described previously [17] to calculate the FDR for the TAB-Seq data and found that although this method yielded similar results for the fetal TAB-Seq data and for cytosines in the CHG and CHH contexts in the adult TAB-Seq data with a FDR <0.05, the FDR was higher than 0.1 even at a *P* value of 1E-8 when applying it to the adult TAB-Seq data. This is likely because the number of *bona fide* hydroxymethylated cytosines of the adult brain is much greater than those of ESCs and the fetal brain. To be consistent, we applied the Benjamini-Hochberg method for both the adult and fetal TAB-Seq data. For calculating the FDR for the BS-Seq data, the Benjamini-Hochberg method yielded a similar result with the method described by Lister *et al.*[29].

### Modification frequency of individual CpG sites

The modC frequency was calculated as dividing modCG by the total coverage according to the BS-Seq data (modCG/CG). The hmCG frequency was similarly calculated according to the TAB-Seq data (hmCG/CG), and the mCG frequency was calculated by subtracting hmCG/CG from modCG/CG.

### Quantification of the hmC and mC levels

The hmC level (%hmC) at a given genomic region or site is calculated by dividing the number of sequenced cytosines by the number of sequenced cytosines plus thymines at this region or site according to the TAB-Seq data, where the reference is in CG context. The mC level (%mC) is calculated by subtracting %hmC from the total modification level (%(hmC + mC)) according to the BS-Seq data.

### Genome annotation

All genomic features were defined based on the HG19 genomic annotation downloaded from the UCSC database. Different genic elements, including transcription start sites (TSS), exons, introns, and transcription terminal sites (TTS), were defined based on the Reference Sequence (RefSeq) database. The CGIs were retrieved from the cpgIslandExt table in UCSC database, and promoter CGIs were defined as overlapping with ± 1 kb of TSS. Repetitive sequences (SINE, LINE, LTR, and Major Satellite) were acquired from the Repeat-Masker track in the UCSC database.

### Calculating enrichment of CpG categories on genomic elements

For a given CpG group (modC^low^, mC^high^, hmC^high^, or Fetal > Adult hmCGs), we counted the fraction of the involved CpG sites at each genomic element as the ‘observed’ distribution value. We also counted the fraction of the overall captured genomic CpG sites at each genomic element as the ‘expected’ distribution value. The fold enrichment value for each genomic element was calculated by dividing the ‘observed’ distribution value by the ‘expected’ distribution value.

### Profiling hmC and mC at the exon-intron boundaries

A total of 176,455 internal exons representing 18,606 genes were retrieved from the RefSeq database, with exclusion of all first last exons and single-exon genes. A total of 12,980 first exons were also retrieved. The average hmC, mC, and modC levels, the hmC/mC ratio, the number of four modCG groups relative to all CpGs, and the CpG sites were calculated for each base ± 150 bp around the exon-intron boundaries. The base composition was also measured ± 10 bp around the 5′ and 3′ splicing sites.

### Calculation of exon inclusion rates

To identify exons that are alternatively spliced, we followed the method described previously [14] with modifications. First, a library of exon-exon junction (EEJ) sequences that comprised all possible exon-exon combination for a gene was generated. Second, RNA-Seq data generated in this study were merged with RNA-Seq data of human cerebral cortex generated previously [48], and then were aligned to the EEJ library using the Bowtie program. Reads mapping to the genomic sequences were discarded before alignment. To determine an EEJ, at least eight mapped nucleotides were needed for each of the two exons. Then, the generated EEJ data were used for calculation of the inclusion value of an exon as ‘% inclusion = 100 × (sum(CiA) + sum(ACj))/((sum(CiA) + sum(ACj) +2 × (sum(CiC2) + sum(C1Cj)))’, where Ci is any possible splicing donor upstream the alternative exon, C1 is the first splicing donor upstream the alternative exon, Cj is any possible splicing acceptor downstream the alternative exon, C2 is the first splicing acceptor downstream the alternative exon, and A indicates the examined exon. A minimum of 10 supporting reads are required and exons without a AG dinucleotides at the 3′splicing sites are omitted. An alternatively spliced exon was defined when the inclusion value was less than 0.8.

### Strand-biased hmCG and mCG profiles on gene bodies

Gene body regions between TSS and TTS were divided into 100 equally sized bins, and average hmC and mC levels were calculated for each bin as well as in 100-bp windows throughout 10 kb upstream and downstream of the gene body. The sense strand and the antisense strand were examined separately. A two-tailed and paired Student’s *t*-Test was applied to determine whether the sense and antisense strand were significantly different from each other.

### Identification of the sense-antisense (SAS) gene paired region

All expressed genes (RPKM ≥1) were divided into two groups according to whether they are transcribed along the positive strand or the negative strand. The SAS gene paired region was identified as the overlap region between these two gene groups.

### Identification of cell type-specific genes and house-keeping genes

Genes enriched in different brain cell types including neurons, astrocytes, and oligodendrocytes were defined according to a previous paper with exclusion of genes that are not expressed in our RNA-Seq data [34]. The house-keeping genes were defined according a previous publication [35].

### Association of hmC and mC profiles with ChIP-Seq data

To profile the distribution of hmC and mC on genomic regions with different histone modifications, we used the recently published genome-wide maps of chromatin states in the adult brain midfrontal lobe [27], including H3K4me1, H3K27ac, H3K4me3, H3K27me3, and H3K9me3.

Enhancers were defined as H3K4me1 peaks that are distant from ± 2.5 kb around any TSS in the switchDbTss table downloaded from the UCSC database. To produce the profiles in Figure 4A, we selected enhancers within intragenic regions, which account for approximately two-thirds of enhancers in the adult human brain [9]. For CGI shores, we selected promoter CGIs. We checked a region ± 5 kb around the center of the H3K4me1 peak and 3 kb upstream and downstream of CGIs. For all profiling, the average hmC, mC, and modC levels, the hmC/mC ratio, and the number of the four modCG groups (hmC^high^, mC^high^, mC^all^, and modC^low^) relative to all CpGs were binned into 50-bp sliding windows in 25-bp steps.

To profile hmC and mC in H3K27me3- and H3K9me3-marked regions, each region was divided into 100 equally sized bins, and average hmC, mC, and modC levels and the hmC/mC ratio were calculated for each bin, as well as in 1-kb windows throughout 10 kb upstream and downstream of the regions.

### Accession numbers

The TAB-Seq and BS-Seq data have been deposited to the NCBI under accession number GSE46710, and the TA-RRBS data have been deposited under accession number GSE55249.

## Competing interests

The authors declare that they have no competing interests.

## Authors’ contributions

LW, JQ, CH, RuiL, and FT conceived and designed the experiments. LW, XianL, LY, RongL, CS, MY, XiaoyuL, YH, HG, XW, JX, YangyuZ, YW, and YanZ performed the experiments. LW, YT, Xiaomeng L, and PZ conducted the bioinformatic analyses of the data. LW, CH, JQ, and FT wrote the paper (with contributions from all of the authors). All authors have read and approved the manuscript for publication.

## Supplementary Material

Additional file 1: Table S1 Summary of sequencing details. Click here for file

Additional file 2: Figure S1 Quantitative values of hmC relative to dC measured by LC-MS/MS. LC-MS/MS was performed to genomic DNAs isolated from several regions of two adult brain and two fetal brain samples. For each sample, the average value with the standard deviation from technical duplicates was shown. Click here for file

Additional file 3: Figure S2 Chromatograms of LC-MS/MS. The LC-MS/MS chromatograms of hmC (left panels) and C (right panels) for genomic DNAs extracted from the adult (a) and fetal (b) frontal lobes were shown with the standard curve (c). The peak area counts are marked. Please go to the figshare website [49] for all the raw chromatograms. Click here for file

Additional file 4: Figure S3 The percentages of hmC (by TAB-Seq) or modC (by BS-Seq) in the fetal hippocampus in the contexts of CG, CHH, and CHG. Click here for file

Additional file 5: Figure S4 Features of hydroxymethylome in the human brain. **(a, b)** Distribution of all covered CpGs according to their hydroxymethylation and methylation frequencies in the adult (a) and the fetal (b) brains. **(c)** Distribution of the CpG categories (modC^low^, mC^high^, and hmC^high^) and all captured CpGs (All CpGs) on different genomic elements. **(d)** Average absolute levels of hmC at different genomic elements in the fetal brain. Click here for file

Additional file 6: Figure S5 Prominent hmC changes at the exon-intron boundaries in the human brain. Profiles of hmC and mC for a 200-bp window (50 bp for exon and 150 bp for intron) around the exon-intron and intron-exon boundaries. Modification levels of hmC, mC, total DNA methylation (hmC + hmC), and the ratio of hmC to mC are shown for all internal exons (*n* = 176,455) in the sense strand. Click here for file

Additional file 7: Table S2 Information of 18,036 exons having CpG sites at 5′ splicing sites. Click here for file

Additional file 8: Figure S6 Profiles of hmC and mC at the exon-intron boundary of exons which have a CpG at 5'ss position +4 or +5. Modification levels of hmC, mC, total DNA methylation (hmC + hmC) were shown for a 40-bp window around the exon-intron boundaries at single-nucleotide resolution of two types of exons, which have a CpG at 5′ss position +4 or +5, and are named +4CG and +5CG exons, respectively. Since a CpG at one position will lead to absence of CpG at the nearest neighboring position and thus no methylation value, we merged the data of the sense and the antisense strands for each type of exons. Click here for file

Additional file 9: Table S3 All expressed genes in the adult human brain revealed by RNA-Seq. Click here for file

Additional file 10: Table S4 hmC, mC, and modC levels on each strand of all individual genes. Click here for file

Additional file 11: Figure S7 The average levels of modC on sense and antisense strands of genes expressed at different levels in the adult brain. One-tailed paired Student’s *t* test. nd, no statistical difference (*P* >0.01). Click here for file

Additional file 12: Figure S8 The mC profiles across each strand of the exon. The profile across exons of sense (lined) and antisense (dotted) strands of highly-expressed genes (red) and no-expression genes (black) in the adult brain showed that a transcription-correlated mC bias toward the antisense strand of the exon. Click here for file

Additional file 13: Figure S9 The mC profiles across each strand of the gene body in the mouse brain. The profile across genes of the sense (lined) and antisense (dotted) strands of expressed genes (*n* = 11,424, red) and genes with no expression (*n* = 5,203, black) in the adult mouse brain showed that a transcription-correlated mC bias toward the antisense strand of the gene body in the mouse brain. The TAB-Seq, BS-Seq, and RNA-Seq data for analysis were obtained from Lister *et al.*[25]. Click here for file

Additional file 14: Figure S10 FACS scatter plot for the control sample for isolation of neuronal nuclei, which was processed with IgG. Click here for file

Additional file 15: Figure S11 Distribution of modC^low^, mC^high^, and hmC^high^ surrounding the midpoints of active or poised enhancers. Click here for file

Additional file 16: Figure S12 hmC and mC maps in a 2-mb genomic region on chromosome 11. This example shows depletion of hmC and enrichment of mC on the H3K9me3- and H3K27me3-marked repressive regions. ChIP-Seq data for H3K4me1, H3K4me3, H3K9me3, and H3K27me3 were obtained from Zhu *et al.*[26]. Click here for file

Additional file 17: Figure S13 hmC and mC maps in a 2.8-mb genomic region surrounding the *NRXN1* gene. This example shows enrichment of hmC and H3K4me1 within the genic region and enrichment of mC and H3K27me3 in the neighboring intergenic regions. ChIP-Seq data for H3K4me1, H3K4me3, H3K9me3, and H3K27me3 were obtained from Zhu *et al.*[26]. Click here for file

## References

[B1] Tahiliani M Koh KP Shen Y Pastor WA Bandukwala H Brudno Y Agarwal S Iyer LM Liu DR Aravind L Rao A Conversion of 5-methylcytosine to 5-hydroxymethylcytosine in mammalian DNA by MLL partner TET1 Science 2009 324 930 935 10.1126/science.1170116 19372391 PMC2715015

[B2] Kriaucionis S Heintz N The nuclear DNA base 5-hydroxymethylcytosine is present in Purkinje neurons and the brain Science 2009 324 929 930 10.1126/science.1169786 19372393 PMC3263819

[B3] He YF Li BZ Li Z Liu P Wang Y Tang Q Ding J Jia Y Chen Z Li L Sun Y Li X Dai Q Song CX Zhang K He C Xu GL Tet-mediated formation of 5-carboxylcytosine and its excision by TDG in mammalian DNA Science 2011 333 1303 1307 10.1126/science.1210944 21817016 PMC3462231

[B4] Ito S Shen L Dai Q Wu SC Collins LB Swenberg JA He C Zhang Y Tet proteins can convert 5-methylcytosine to 5-formylcytosine and 5-carboxylcytosine Science 2011 333 1300 1303 10.1126/science.1210597 21778364 PMC3495246

[B5] Wu H D’Alessio AC Ito S Wang Z Cui K Zhao K Sun YE Zhang Y Genome-wide analysis of 5-hydroxymethylcytosine distribution reveals its dual function in transcriptional regulation in mouse embryonic stem cells Genes Dev 2011 25 679 684 10.1101/gad.2036011 21460036 PMC3070931

[B6] Williams K Christensen J Pedersen MT Johansen JV Cloos PA Rappsilber J Helin K TET1 and hydroxymethylcytosine in transcription and DNA methylation fidelity Nature 2011 473 343 348 10.1038/nature10066 21490601 PMC3408592

[B7] Pastor WA Pape UJ Huang Y Henderson HR Lister R Ko M McLoughlin EM Brudno Y Mahapatra S Kapranov P Tahiliani M Daley GQ Liu XS Ecker JR Milos PM Agarwal S Rao A Genome-wide mapping of 5-hydroxymethylcytosine in embryonic stem cells Nature 2011 473 394 397 10.1038/nature10102 21552279 PMC3124347

[B8] Song CX Szulwach KE Fu Y Dai Q Yi C Li X Li Y Chen CH Zhang W Jian X Wang J Zhang L Looney TJ Zhang B Godley LA Hicks LM Lahn BT Jin P He C Selective chemical labeling reveals the genome-wide distribution of 5-hydroxymethylcytosine Nat. Biotechnol 2011 29 68 72 10.1038/nbt.1732 21151123 PMC3107705

[B9] Szulwach KE Li X Li Y Song CX Wu H Dai Q Irier H Upadhyay AK Gearing M Levey AI Vasanthakumar A Godley LA Chang Q Cheng X He C Jin P 5-hmC-mediated epigenetic dynamics during postnatal neurodevelopment and aging Nat. Neurosci 2011 14 1607 1616 10.1038/nn.2959 22037496 PMC3292193

[B10] Ficz G Branco MR Seisenberger S Santos F Krueger F Hore TA Marques CJ Andrews S Reik W Dynamic regulation of 5-hydroxymethylcytosine in mouse ES cells and during differentiation Nature 2011 473 398 402 10.1038/nature10008 21460836

[B11] Stroud H Feng S Morey Kinney S Pradhan S Jacobsen SE 5-Hydroxymethylcytosine is associated with enhancers and gene bodies in human embryonic stem cells Genome Biol 2011 12 R54 10.1186/gb-2011-12-6-r54 21689397 PMC3218842

[B12] Xu Y Wu F Tan L Kong L Xiong L Deng J Barbera AJ Zheng L Zhang H Huang S Min J Nicholson T Chen T Xu G Shi Y Zhang K Shi YG Genome-wide regulation of 5hmC, 5mC, and gene expression by Tet1 hydroxylase in mouse embryonic stem cells Mol. Cell 2011 42 451 464 10.1016/j.molcel.2011.04.005 21514197 PMC3099128

[B13] Jin SG Wu X Li AX Pfeifer GP Genomic mapping of 5-hydroxymethylcytosine in the human brain Nucleic Acids Res 2011 39 5015 5024 10.1093/nar/gkr120 21378125 PMC3130285

[B14] Khare T Pai S Koncevicius K Pal M Kriukiene E Liutkeviciute Z Irimia M Jia P Ptak C Xia M Tice R Tochigi M Moréra S Nazarians A Belsham D Wong AH Blencowe BJ Wang SC Kapranov P Kustra R Labrie V Klimasauskas S Petronis A 5-hmC in the brain is abundant in synaptic genes and shows differences at the exon-intron boundary Nat Struct Mol Biol 2012 19 1037 1043 10.1038/nsmb.2372 22961382 PMC3465469

[B15] Sun Z Terragni J Borgaro JG Liu Y Yu L Guan S Wang H Sun D Cheng X Zhu Z Pradhan S Zheng Y High-resolution enzymatic mapping of genomic 5-hydroxymethylcytosine in mouse embryonic stem cells Cell Rep 2013 3 567 576 10.1016/j.celrep.2013.01.001 23352666 PMC3743234

[B16] Gan H Wen L Liao S Lin X Ma T Liu J Song CX Wang M He C Han C Tang F Dynamics of 5-hydroxymethylcytosine during mouse spermatogenesis Nat Commun 2013 4 1995 23759713 10.1038/ncomms2995

[B17] Yu M Hon GC Szulwach KE Song CX Zhang L Kim A Li X Dai Q Shen Y Park B Min JH Jin P Ren B He C Base-resolution analysis of 5-hydroxymethylcytosine in the mammalian genome Cell 2012 149 1368 1380 10.1016/j.cell.2012.04.027 22608086 PMC3589129

[B18] Booth MJ Branco MR Ficz G Oxley D Krueger F Reik W Balasubramanian S Quantitative sequencing of 5-methylcytosine and 5-hydroxymethylcytosine at single-base resolution Science 2012 336 934 937 10.1126/science.1220671 22539555

[B19] Globisch D Münzel M Müller M Michalakis S Wagner M Koch S Brückl T Biel M Carell T Tissue distribution of 5-hydroxymethylcytosine and search for active demethylation intermediates PLoS ONE 2010 5 e15367 10.1371/journal.pone.0015367 21203455 PMC3009720

[B20] Guo JU Su Y Zhong C Ming GL Song H Hydroxylation of 5-methylcytosine by TET1 promotes active DNA demethylation in the adult brain Cell 2011 145 423 434 10.1016/j.cell.2011.03.022 21496894 PMC3088758

[B21] Mellén M Ayata P Dewell S Kriaucionis S Heintz N MeCP2 binds to 5hmC enriched within active genes and accessible chromatin in the nervous system Cell 2012 151 1417 1430 10.1016/j.cell.2012.11.022 23260135 PMC3653293

[B22] Spruijt CG Gnerlich F Smits AH Pfaffeneder T Jansen PW Bauer C Münzel M Wagner M Müller M Khan F Eberl HC Mensinga A Brinkman AB Lephikov K Müller U Walter J Boelens R van Ingen H Leonhardt H Carell T Vermeulen M Dynamic readers for 5-(hydroxy)methylcytosine and its oxidized derivatives Cell 2013 152 1146 1159 10.1016/j.cell.2013.02.004 23434322

[B23] Zhang RR Cui QY Murai K Lim YC Smith ZD Jin S Ye P Rosa L Lee YK Wu HP Liu W Xu ZM Yang L Ding YQ Tang F Meissner A Ding C Shi Y Xu GL et1 regulates adult hippocampal neurogenesis and cognition Cell Stem Cell 2013 13 237 45 10.1016/j.stem.2013.05.006 23770080 PMC4474382

[B24] Rudenko A Dawlaty MM Seo J Cheng AW Meng J Le T Faull KF Jaenisch R Tsai LH Tet1 is critical for neuronal activity-regulated gene expression and memory extinction Neuron 2013 79 1109 22 10.1016/j.neuron.2013.08.003 24050401 PMC4543319

[B25] Lister R Mukamel EA Nery JR Urich M Puddifoot CA Johnson ND Lucero J Huang Y Dwork AJ Schultz MD Yu M Tonti-Filippini J Heyn H Hu S Wu JC Rao A Esteller M He C Haghighi FG Sejnowski TJ Behrens MM Ecker JR Global epigenomic reconfiguration during mammalian brain development Science 2013 341 1237905 10.1126/science.1237905 23828890 PMC3785061

[B26] Stadler MB Murr R Burger L Ivanek R Lienert F Schöler A van Nimwegen E Wirbelauer C Oakeley EJ Gaidatzis D Tiwari VK Schübeler D DNA-binding factors shape the mouse methylome at distal regulatory regions Nature 2011 480 490 495 22170606 10.1038/nature10716

[B27] Zhu J Adli M Zou JY Verstappen G Coyne M Zhang X Durham T Miri M Deshpande V De Jager PL Bennett DA Houmard JA Muoio DM Onder TT Camahort R Cowan CA Meissner A Epstein CB Shoresh N Bernstein BE Genome-wide chromatin state transitions associated with developmental and environmental cues Cell 2013 152 642 654 10.1016/j.cell.2012.12.033 23333102 PMC3563935

[B28] Creyghton MP Cheng AW Welstead GG Kooistra T Carey BW Steine EJ Hanna J Lodato MA Frampton GM Sharp PA Boyer LA Young RA Jaenisch R Histone H3K27ac separates active from poised enhancers and predicts developmental state Proc Natl Acad Sci U S A 2010 107 21931 21936 10.1073/pnas.1016071107 21106759 PMC3003124

[B29] Lister R Pelizzola M Dowen RH Hawkins RD Hon G Tonti-Filippini J Nery JR Lee L Ye Z Ngo QM Edsall L Antosiewicz-Bourget J Stewart R Ruotti V Millar AH Thomson JA Ren B Ecker JR Human DNA methylomes at base resolution show widespread epigenomic differences Nature 2009 462 315 322 10.1038/nature08514 19829295 PMC2857523

[B30] Laurent L Wong E Li G Huynh T Tsirigos A Ong CT Low HM Kin Sung KW Rigoutsos I Loring J Wei CL Dynamic changes in the human methylome during differentiation Genome Res 2009 20 320 331 20133333 10.1101/gr.101907.109 PMC2840979

[B31] Chodavarapu RK Feng S Bernatavichute YV Chen PY Stroud H Yu Y Hetzel JA Kuo F Kim J Cokus SJ Casero D Bernal M Huijser P Clark AT Krämer U Merchant SS Zhang X Jacobsen SE Pellegrini M Relationship between nucleosome positioning and DNA methylation Nature 2010 466 388 392 10.1038/nature09147 20512117 PMC2964354

[B32] Gelfman S Cohen N Yearim A Ast G DNA-methylation effect on cotranscriptional splicing is dependent on GC architecture of the exon-intron structure Genome Res 2013 23 789 799 10.1101/gr.143503.112 23502848 PMC3638135

[B33] Maunakea AK Chepelev I Cui K Zhao K Intragenic DNA methylation modulates alternative splicing by recruiting MeCP2 to promote exon recognition Cell Res 2013 23 1256 1269 10.1038/cr.2013.110 23938295 PMC3817542

[B34] Cahoy JD Emery B Kaushal A Foo LC Zamanian JL Christopherson KS Xing Y Lubischer JL Krieg PA Krupenko SA Thompson WJ Barres BA A transcriptome database for astrocytes, neurons, and oligodendrocytes: a new resource for understanding brain development and function J Neurosci 2008 28 264 278 10.1523/JNEUROSCI.4178-07.2008 18171944 PMC6671143

[B35] Eisenberg E Levanon EY Human housekeeping genes, revisited Trends Genet 2013 29 569 574 10.1016/j.tig.2013.05.010 23810203

[B36] Braunschweig U Gueroussov S Plocik AM Graveley BR Blencowe BJ Dynamic integration of splicing within gene regulatory pathways Cell 2013 152 1252 1269 10.1016/j.cell.2013.02.034 23498935 PMC3642998

[B37] Hellman A Chess A Gene body-specific methylation on the active X chromosome Science 2007 315 1141 1143 10.1126/science.1136352 17322062

[B38] Zhang X Yazaki J Sundaresan A Cokus S Chan SW Chen H Henderson IR Shinn P Pellegrini M Jacobsen SE Ecker JR Genome-wide high-resolution mapping and functional analysis of DNA methylation in *arabidopsis* Cell 2006 126 1189 1201 10.1016/j.cell.2006.08.003 16949657

[B39] Zemach A McDaniel IE Silva P Zilberman D Genome-wide evolutionary analysis of eukaryotic DNA methylation Science 2010 328 916 919 10.1126/science.1186366 20395474

[B40] Jones PA Functions of DNA methylation: islands, start sites, gene bodies and beyond Nat Rev Genet 2012 13 484 492 10.1038/nrg3230 22641018

[B41] Ball MP Li JB Gao Y Lee JH LeProust EM Park IH Xie B Daley GQ Church GM Targeted and genome-scale strategies reveal gene-body methylation signatures in human cells Nat Biotechnol 2009 27 361 368 10.1038/nbt.1533 19329998 PMC3566772

[B42] Rauch TA Wu X Zhong X Riggs AD Pfeifer GP A human B cell methylome at 100-base pair resolution Proc Natl Acad Sci U S A 2009 106 671 678 10.1073/pnas.0812399106 19139413 PMC2621253

[B43] Bird AP Gene number, noise reduction and biological complexity Trends Genet 1995 11 94 100 10.1016/S0168-9525(00)89009-5 7732579

[B44] Yu M Hon GC Szulwach KE Song CX Jin P Ren B He C Tet-assisted bisulfite sequencing of 5-hydroxymethylcytosine Nat Protoc 2012 7 2159 2170 10.1038/nprot.2012.137 23196972 PMC3641661

[B45] Gu H Smith ZD Bock C Boyle P Gnirke A Meissner A Preparation of reduced representation bisulfite sequencing libraries for genome-scale DNA methylation profiling Nat Protoc 2011 6 468 481 10.1038/nprot.2010.190 21412275

[B46] Gu H Smith ZD Bock C Boyle P Gnirke A Meissner A Isolation of neuronal chromatin from brain tissue BMC Neurosci 2008 9 42 10.1186/1471-2202-9-42 18442397 PMC2377267

[B47] Benjamini Y Hochberg Y Controlling the false discovery rate: a practical and powerful approach to multiple testing J Roy Stat Soc B Met 1995 57 289 300

[B48] Brawand D Soumillon M Necsulea A Julien P Csardi G Harrigan P Weier M Liechti A Aximu-Petri A Kircher MM Albert FW Zeller U Khaitovich P Grützner F Bergmann S Nielsen R Pääbo S Kaessmann H The evolution of gene expression levels in mammalian organs Nature 2011 478 343 348 10.1038/nature10532 22012392

[B49] The LC-MS/MS chromatograms of hmC and C for genomic DNAs extracted from the adult and fetal brains http://dx.doi.org/10.6084/m9.figshare.943481

