# Supplementary figures and images for: Whole-genome analysis of 5-hydroxymethylcytosine and 5-methylcytosine at base resolution in the human brain
Source: Genome Biol. 2014 Mar 4;15(3):R49. doi: 10.1186/gb-2014-15-3-r49 (PMC4053808; doi:10.1186/gb-2014-15-3-r49)

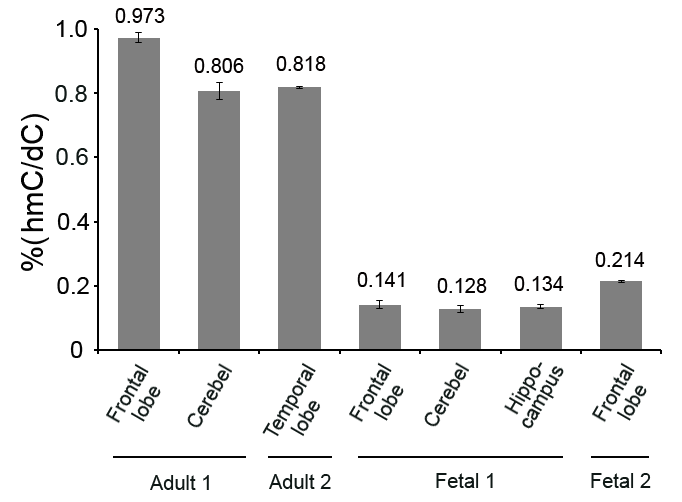

Supplement: Additional file 2: Figure S1 — Quantitative values of hmC relative to dC measured by LC-MS/MS. LC-MS/MS was performed to genomic DNAs isolated from several regions of two adult brain and two fetal brain samples. For each sample, the average value with the standard deviation from technical duplicates was shown. [file gb-2014-15-3-r49-S2.tiff]

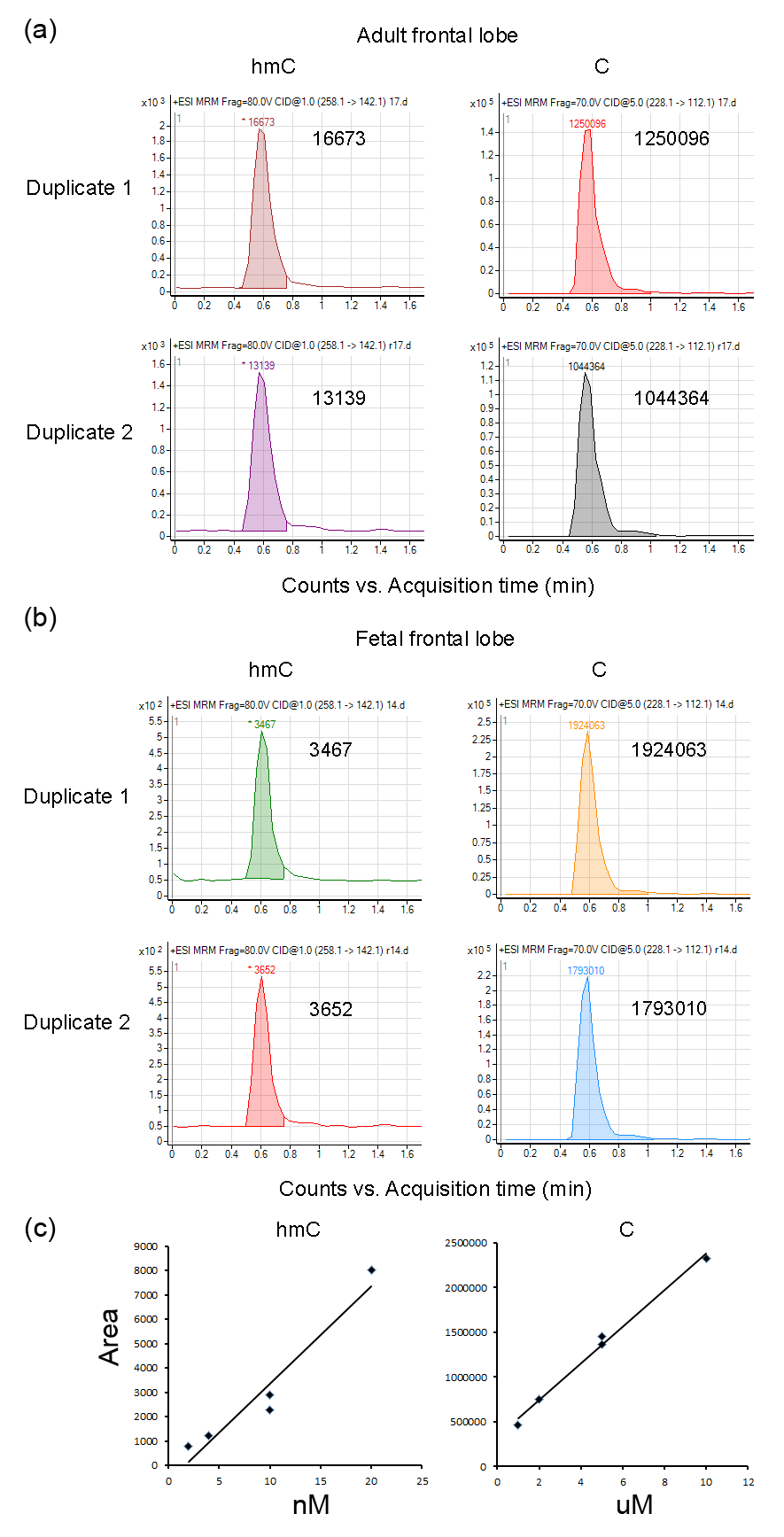

Supplement: Additional file 3: Figure S2 — Chromatograms of LC-MS/MS. The LC-MS/MS chromatograms of hmC (left panels) and C (right panels) for genomic DNAs extracted from the adult (a) and fetal (b) frontal lobes were shown with the standard curve (c). The peak area counts are marked. Please go to the figshare website [49] for all the raw chromatograms. [file gb-2014-15-3-r49-S3.tiff]

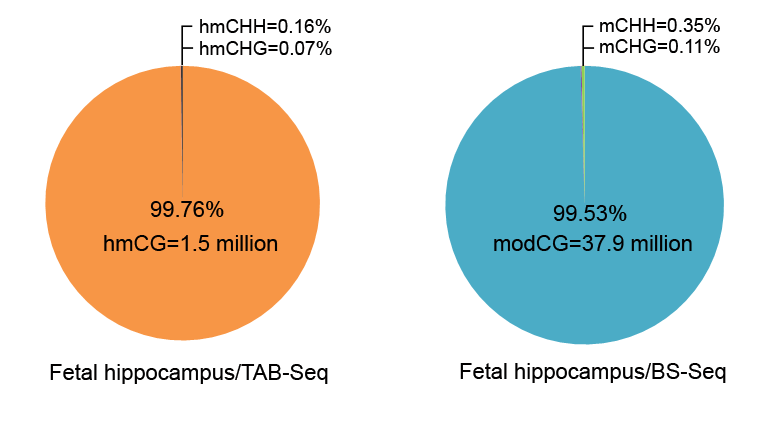

Supplement: Additional file 4: Figure S3 — The percentages of hmC (by TAB-Seq) or modC (by BS-Seq) in the fetal hippocampus in the contexts of CG, CHH, and CHG. [file gb-2014-15-3-r49-S4.tiff]

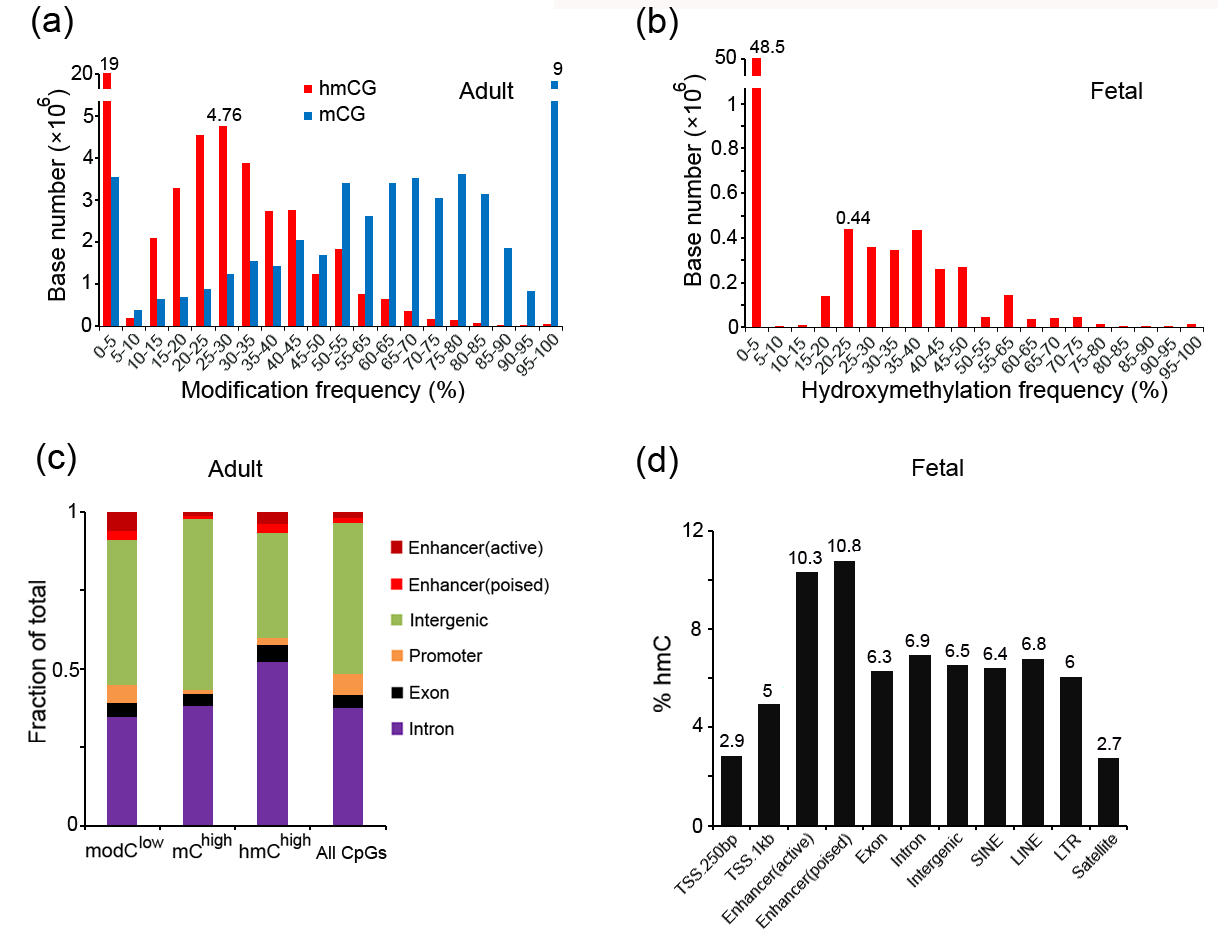

Supplement: Additional file 5: Figure S4 — Features of hydroxymethylome in the human brain. (a, b) Distribution of all covered CpGs according to their hydroxymethylation and methylation frequencies in the adult (a) and the fetal (b) brains. (c) Distribution of the CpG categories (modClow, mChigh, and hmChigh) and all captured CpGs (All CpGs) on different genomic elements. (d) Average absolute levels of hmC at different genomic elements in the fetal brain. [file gb-2014-15-3-r49-S5.tiff]

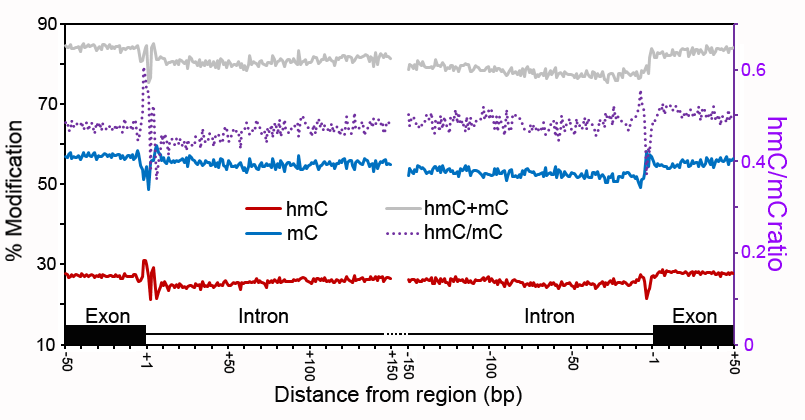

Supplement: Additional file 6: Figure S5 — Prominent hmC changes at the exon-intron boundaries in the human brain. Profiles of hmC and mC for a 200-bp window (50 bp for exon and 150 bp for intron) around the exon-intron and intron-exon boundaries. Modification levels of hmC, mC, total DNA methylation (hmC + hmC), and the ratio of hmC to mC are shown for all internal exons (n = 176,455) in the sense strand. [file gb-2014-15-3-r49-S6.tiff]

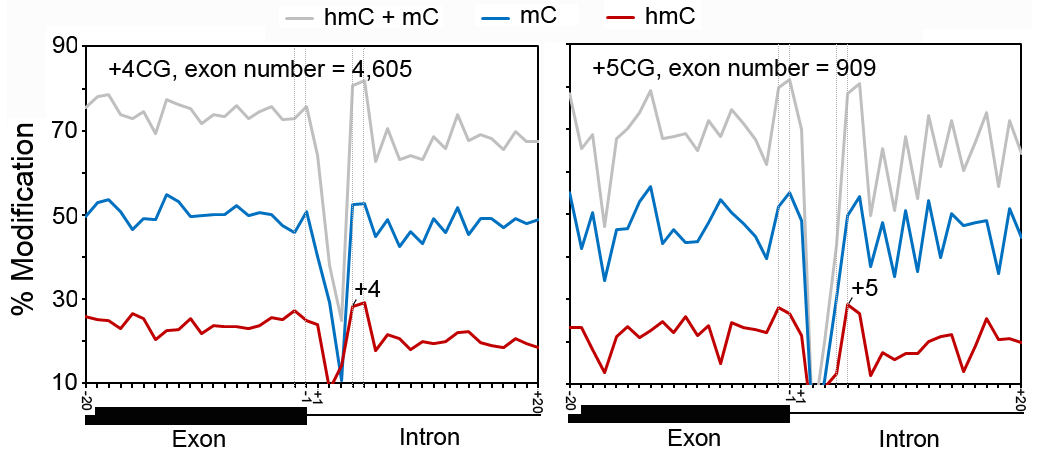

Supplement: Additional file 8: Figure S6 — Profiles of hmC and mC at the exon-intron boundary of exons which have a CpG at 5'ss position +4 or +5. Modification levels of hmC, mC, total DNA methylation (hmC + hmC) were shown for a 40-bp window around the exon-intron boundaries at single-nucleotide resolution of two types of exons, which have a CpG at 5′ss position +4 or +5, and are named +4CG and +5CG exons, respectively. Since a CpG at one position will lead to absence of CpG at the nearest neighboring position and thus no methylation value, we merged the data of the sense and the antisense strands for each type of exons. [file gb-2014-15-3-r49-S8.tiff]

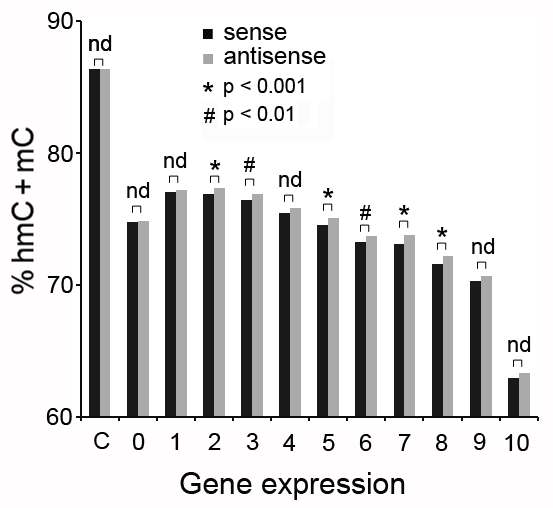

Supplement: Additional file 11: Figure S7 — The average levels of modC on sense and antisense strands of genes expressed at different levels in the adult brain. One-tailed paired Student’s t test. nd, no statistical difference (P >0.01). [file gb-2014-15-3-r49-S11.tiff]

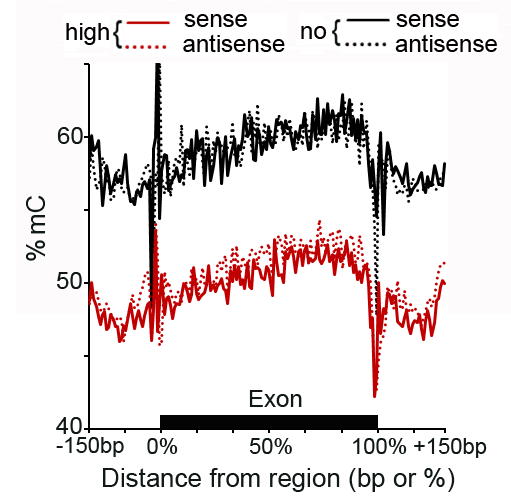

Supplement: Additional file 12: Figure S8 — The mC profiles across each strand of the exon. The profile across exons of sense (lined) and antisense (dotted) strands of highly-expressed genes (red) and no-expression genes (black) in the adult brain showed that a transcription-correlated mC bias toward the antisense strand of the exon. [file gb-2014-15-3-r49-S12.tiff]

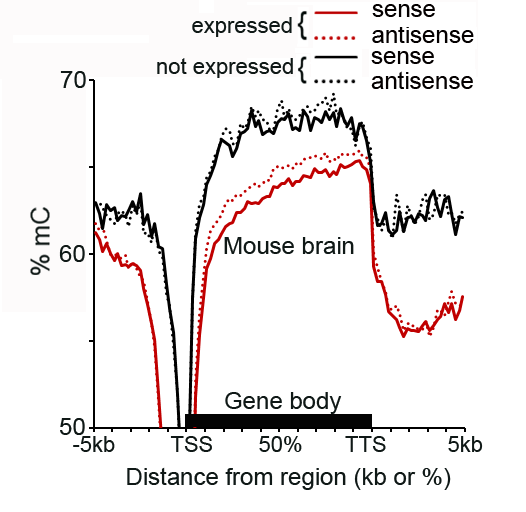

Supplement: Additional file 13: Figure S9 — The mC profiles across each strand of the gene body in the mouse brain. The profile across genes of the sense (lined) and antisense (dotted) strands of expressed genes (n = 11,424, red) and genes with no expression (n = 5,203, black) in the adult mouse brain showed that a transcription-correlated mC bias toward the antisense strand of the gene body in the mouse brain. The TAB-Seq, BS-Seq, and RNA-Seq data for analysis were obtained from Lister et al.[25]. [file gb-2014-15-3-r49-S13.tiff]

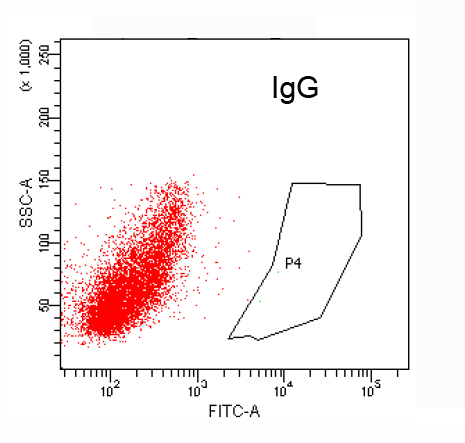

Supplement: Additional file 14: Figure S10 — FACS scatter plot for the control sample for isolation of neuronal nuclei, which was processed with IgG. [file gb-2014-15-3-r49-S14.tiff]

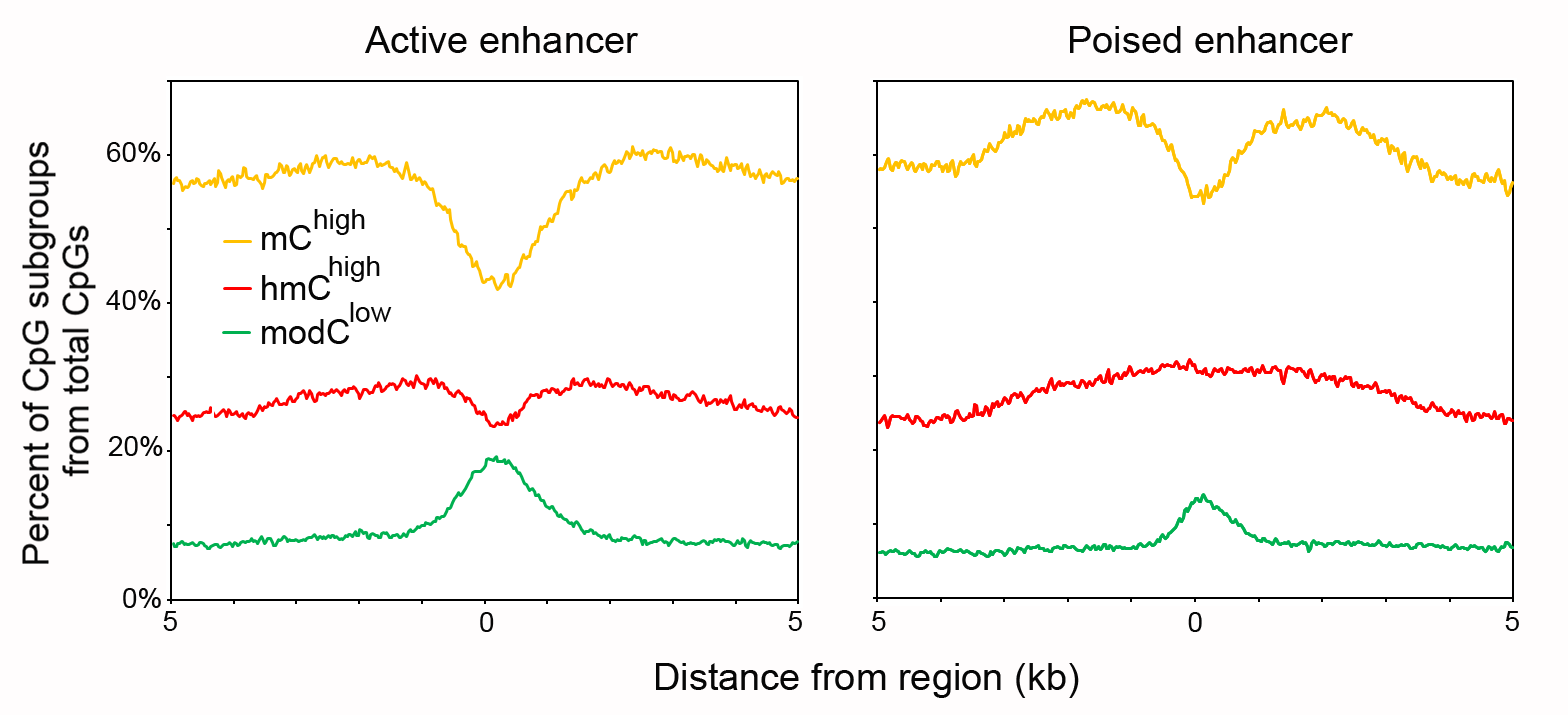

Supplement: Additional file 15: Figure S11 — Distribution of modClow, mChigh, and hmChigh surrounding the midpoints of active or poised enhancers. [file gb-2014-15-3-r49-S15.tiff]

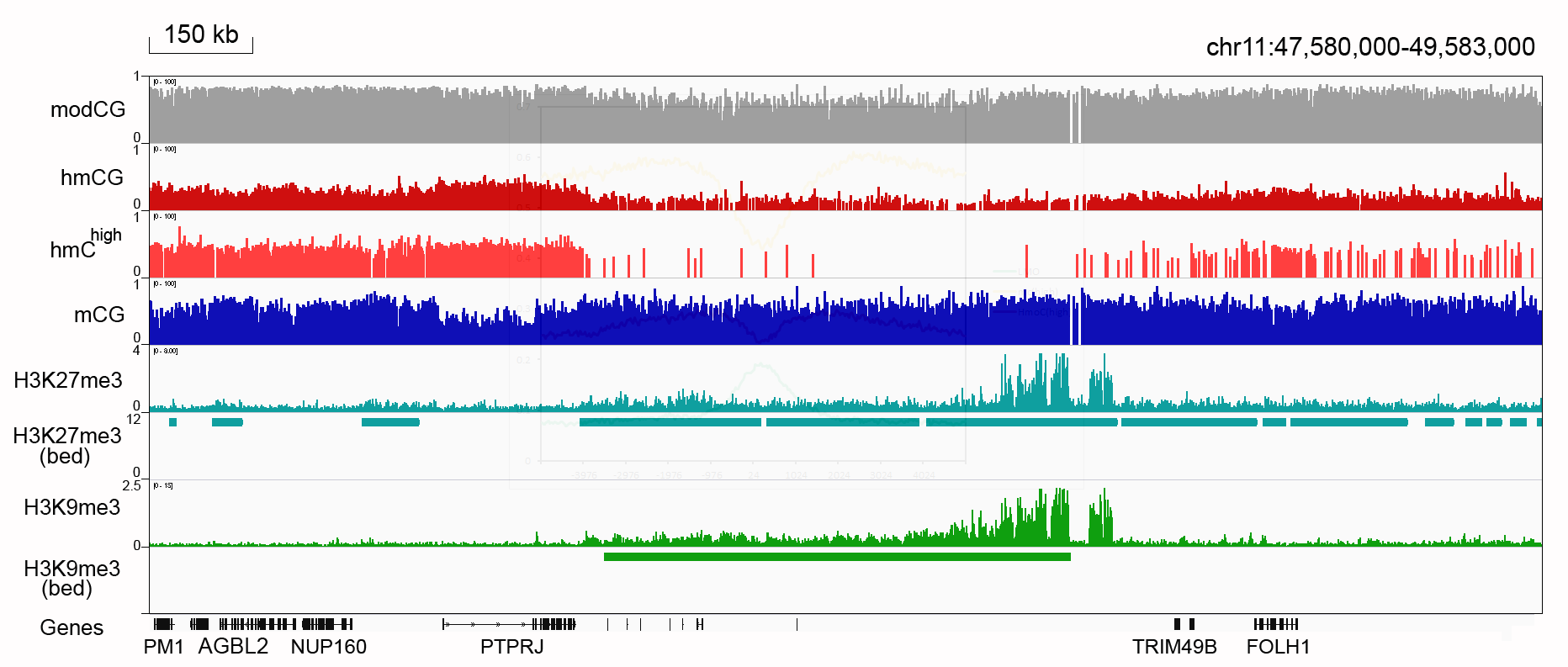

Supplement: Additional file 16: Figure S12 — hmC and mC maps in a 2-mb genomic region on chromosome 11. This example shows depletion of hmC and enrichment of mC on the H3K9me3- and H3K27me3-marked repressive regions. ChIP-Seq data for H3K4me1, H3K4me3, H3K9me3, and H3K27me3 were obtained from Zhu et al.[26]. [file gb-2014-15-3-r49-S16.tiff]

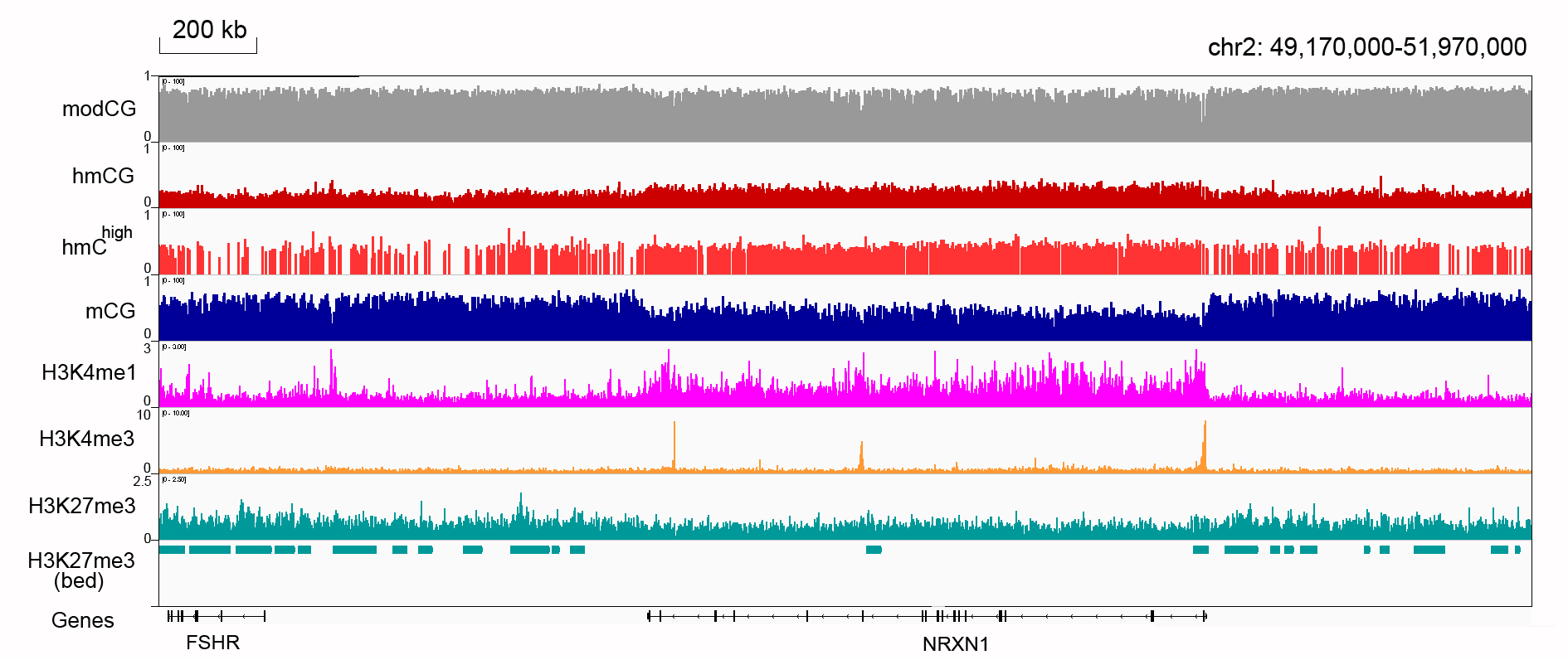

Supplement: Additional file 17: Figure S13 — hmC and mC maps in a 2.8-mb genomic region surrounding the NRXN1 gene. This example shows enrichment of hmC and H3K4me1 within the genic region and enrichment of mC and H3K27me3 in the neighboring intergenic regions. ChIP-Seq data for H3K4me1, H3K4me3, H3K9me3, and H3K27me3 were obtained from Zhu et al.[26]. [file gb-2014-15-3-r49-S17.tiff]
